# *In situ* molecular imaging of adsorbed protein films in water indicating hydrophobicity and hydrophilicity

**DOI:** 10.1038/s41598-020-60428-1

**Published:** 2020-02-28

**Authors:** Jiachao Yu, Yufan Zhou, Mark Engelhard, Yuchen Zhang, Jiyoung Son, Songqin Liu, Zihua Zhu, Xiao-Ying Yu

**Affiliations:** 1 Jiangsu Province Hi-Tech Key Laboratory for Bio-medical Research, School of Chemistry and Chemical Engineering, Southeast University, Nanjing, 210096 China; 2 Energy and Environment Directorate, Pacific Northwest National Laboratory, Richland, WA 99354 USA; 3 Environmental Molecular Sciences Laboratory, Pacific Northwest National Laboratory, Richland, WA 99354 USA; 4 Department of Chemistry, School of Science, Zhejiang Sci-Tech University, Hangzhou, 310018 China

**Keywords:** Biosurfaces, Biomaterials - proteins

## Abstract

*In situ* molecular imaging of protein films adsorbed on a solid surface in water was realized by using a vacuum compatible microfluidic interface and time-of-flight secondary ion mass spectrometry (ToF-SIMS). Amino acid fragments from such hydrated protein films are observed and identified in the positive ion mode and the results are in agreement with reported works on dry protein films. Moreover, water clusters from the hydrated protein films have been observed and identified in both the positive and negative ion mode for a series protein films. Thus, the detailed composition of amino acids and water molecules in the hydrated protein films can be characterized, and the protein water microstructures can be revealed by the distinct three-dimensional spatial distribution reconstructed from *in situ* liquid ToF-SIMS molecular imaging. Furthermore, spectral principal component analysis of amino acid fragment peaks and water cluster peaks provides unique insights into the water cluster distribution, hydrophilicity, and hydrophobicity of hydrated adsorbed protein films in water.

## Introduction

Hydration is crucial to keep the conformation and biological activity of proteins^1,2^. It is well-known that water molecules around proteins have properties quite distinct from those in the aqueous bulk phase^3,4^. As a representative form of hydrated proteins, the hydrated protein films consist of a mixed layer of protein and water molecules^5,6^. Thus, the revelation of the composition and structure of hydrated protein films, especially for the distribution of water molecules within them, is an effective way to understand the hydration mechanism of proteins.

Adsorbed protein films on a solid surface have been widely studied because of their unique feature in cell adhesion, which has multiple applications in biology and medicine, such as cell culture^7^ and implantable device manufacture^8^. The composition and structure of adsorbed hydrated protein films are usually evaluated by analyzing their dry samples due to the technical and instrumental limitations to study the solid-liquid interface using surface sensitive techniques that are largely constrained because of vacuum conditions^9,10^. The orientation of the adsorbed protein molecules is of great interest^9,10^. However, the dry protein film is rather different from the hydrated protein film in its natural state. While dry sample surface analysis is useful to provide ensemble measurements of the chemical composition, thickness, and possibly orientation, they do not necessarily reflect the composition and structure of hydrated protein films in water. More importantly, the water microenvironment surrounding the protein and water molecules associated with the protein structure are lost in dry sample analysis. Actually, as far as we know, few experimental techniques can be used to analyze the water microenvironment surrounding the protein molecules, and most understandings in this field have been from computational efforts^1,11,12^. Therefore, direct molecular evidence is of great importance to verify theoretical calculations.

**Figure 1:**
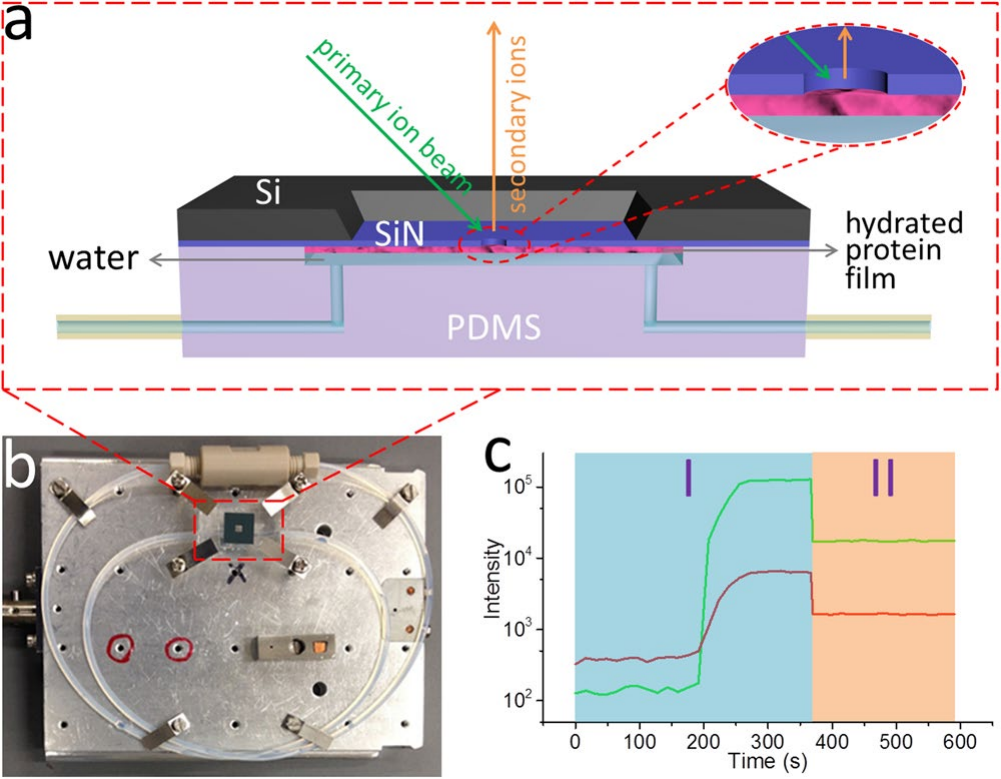
(**a**) Scheme of hydrated protein film attached to the SiN membrane in a SALVI device during ToF-SIMS measurement. (**b**) A photo of SALVI device assembled on a ToF-SIMS stage. (**c**) ToF-SIMS profiles of an amino acid fragment (CH_5_N_3_^+^, m/z 59, red curve) and a water cluster ((H_2_O)_3_H^+^, m/z 55, green curve) from the adsorbed and hydrated BSA film during two measuring periods: using the Bi_3_^+ ^primary ion beam with (I) long pulse width and (II) short pulse width.

A microfluidic device, System for Analysis at the Liquid Vacuum Interface (SALVI), was developed to enable direct surface analysis of liquid surfaces and solid-liquid interfaces using vacuum-based surface tools including Time-of-Flight Secondary Ion Mass Spectrometry (ToF-SIMS)^13^. *In situ* liquid ToF-SIMS enabled by SALVI was employed to probe the adsorbed hydrated protein film on a solid surface. In this paper, we investigated five representative hydrated protein films and pure water using *in situ* molecular imaging. Briefly, the hydrated protein film was immobilized on the SiN surface of the microchannel (Fig. 1a), which was then assembled on the ToF-SIMS stage followed with *in situ* analysis (Fig. 1b). A representative liquid ToF-SIMS depth profile of a hydrated protein film is shown in Fig. 1c. The SIMS m/z spectra and 3D images have provided a detailed evaluation on the composition and structure of these hydrated adsorbed protein films for the first time.

## Results

The effect of the primary ion beam on water clusters and its potential implications in biological analysis were discussed in earlier works^14,15^. The Bi_3_^+^ primary ion beam was used to obtain optical results of organics in this work. Water is the most likely source of the matrix effect in this study. DI water is included in the spectral analysis and principal component analysis (PCA). These results show that water clusters from the bulk water are different from those observed in the protein monolayers formed in DI water.

The matrix effect has long been a concern in ToF-SIMS analysis^16^. ToF-SIMS signal intensity can vary in several orders of magnitude due to the matrix affect. Therefore, absolute quantification has been challenging. In fact, this is an important reason that ToF-SIMS has not been a popular analysis tool, though it was developed more than 50 years ago. However, semi-quantification (i.e., relative concentrations among samples can be obtained based on comparison of SIMS spectra from various samples) is possible if their matrices are similar. This approach has been widely used in ToF-SIMS analysis^17^. In other words, multiple relevant samples of the same matrix are analysed to reduce the known effect in a sample matrix instead of individually as in other more quantitative techniques. Because all protein films form in water, it is reasonable to expect that the effect of the matrix can be minimized when comparing *in situ* liquid ToF-SIMS data from different protein films in data analysis. PCA is a data analysis strategy based on comparison of spectra, and it is suitable for SIMS analysis^18^. Furthermore, the water matrix in this work has an extra advantage, because a recent study suggests that water molecules can act as a proton source to enhance positive ion yields in SIMS analysis of biological and organic samples^19^.

### Identification of water clusters from protein films

**Figure 2:**
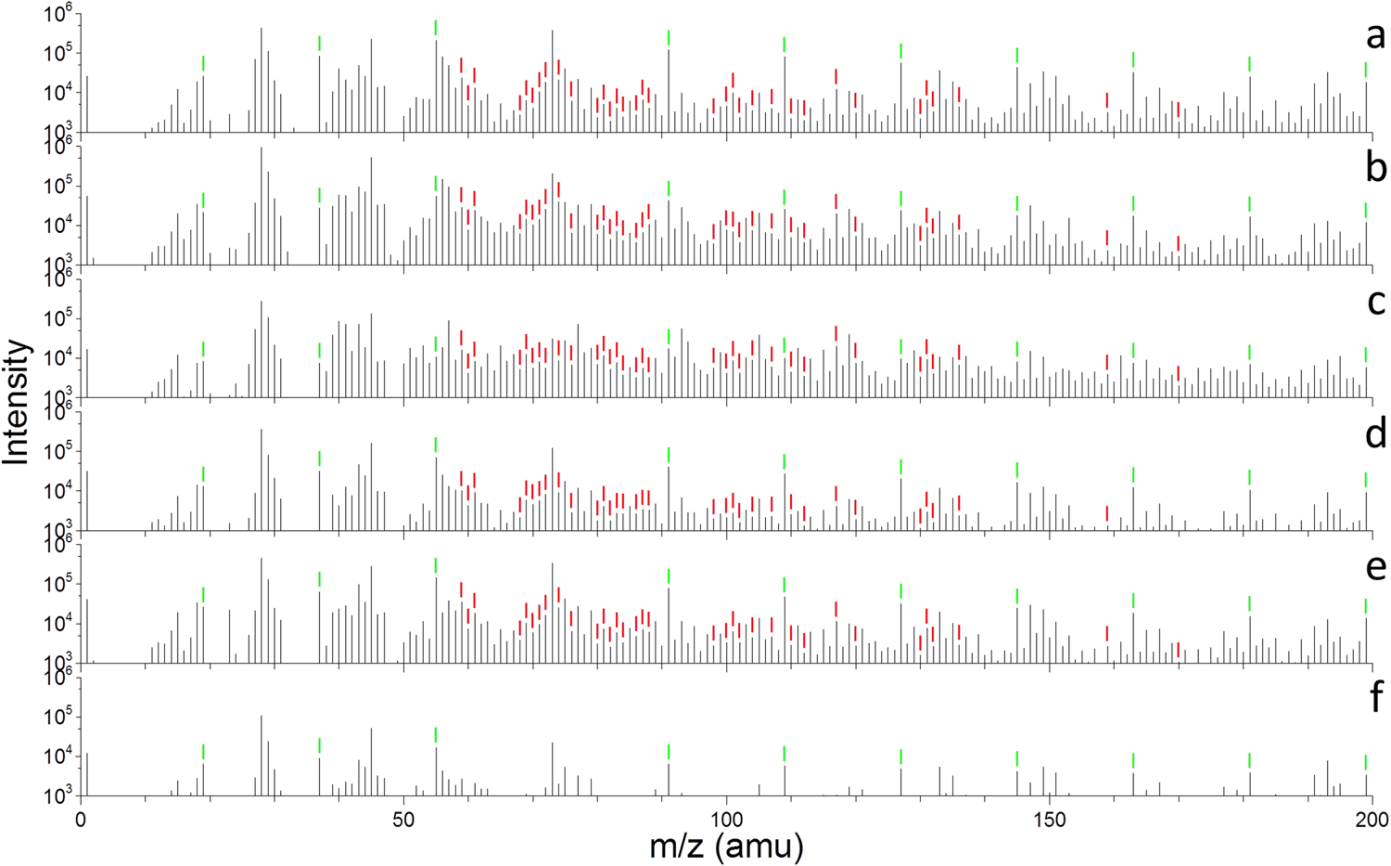
ToF-SIMS spectra (m/z 1–200) of six samples: (**a**) BSA, (b) collagen, (**c**) fibronectin, (**d**) laminin, (**e**) vitronectin, and (**f**) water in the positive ion mode. Amino acid fragments were marked with red labels and water clusters were marked with green labels.

The ToF-SIMS spectra with higher mass resolution were reconstructed from the period II (200 s) in Fig. 1c^20^. Fig. 2 shows representative liquid ToF-SIMS spectra (m/z 1–200) of BSA, collagen, fibronectin, laminin, vitronectin and water in the positive ion mode. Additional positive spectra and negative spectra are depicted in Figs. S1–S3, respectively. In the positive ion mode, water cluster peaks are marked with green labels in Fig. 2 and S2. Except for (H_2_O)_4_H^+^, m/z 73 unresolved from a polydimethylsiloxane (PDMS) fragment interference peak (SiC_3_H_9_^+^)^21^, a group of positive water cluster peaks ((H_2_O)_n_H^+^, n = 1–41, m/z 19–739) are clearly observed. Similarly, in the negative ion mode (Figs. S1 and S3), a group of negative water cluster peaks ((H_2_O)_n_OH^−^, n = 1–40, m/z 35–737) is observed except for (H_2_O)_10_OH^−^, m/z 197. The latter is not resolved from a silicon oxide cluster interference peak ((SiO_2_)_3_OH^−^)^22^ due to the unit mass resolution used in liquid SIMS^23^.

The positive and negative water clusters are summarized in Table S1. This has been the first comparison of water clusters from adsorbed aqueous protein films using the SALVI and *in situ* liquid ToF-SIMS approach, to the best of our knowledge. Figure 2 and S1 show that the intensities of water clusters from hydrated protein films are higher than that from pure water in both the positive and negative ion mode, verifying that the water molecules bonded in hydrated protein films have different properties and intermolecular interactions compared with those in pure water^3,4^. Meanwhile, the relative intensity of any given water cluster also varies among different hydrated protein films, which suggests that the local microenvironment and distribution of water molecules depend on the hydrophilicity, hydrophobicity, and the structure of the hydrated protein film^12,24^.

Although amino acid fragments are seen in ToF-SIMS spectra, their counts are much lower than those of water clusters in the positive ion mode. This observation is very interesting, because only amino acid fragments were reported in the ToF-SIMS analysis of dry protein samples in the past. Our results show that water clusters are quite important in defining the protein structures and functions in water given their high counts and frequent occurrences.

### Identification of amino acid fragments from protein films

The amino acid fragment peaks in the positive ion mode are observed and marked with red labels in Fig. 2a–e. These amino acid fragments are identified by comparing the spectra of the hydrated protein films with those of corresponding dry protein samples as seen in Fig. S4 and the reported amino acid fragments from dry protein samples using ToF-SIMS^9,10^. The amino acid fragments in liquid ToF-SIMS are depicted in Fig. S4b. These fragments are observed and checked against reference samples in the dry protein samples, and peak identification is summarized in Table S2. The spectral comparison shows that peak identification obtained in the liquid SIMS with lower mass accuracy is in agreement with those obtained using high mass resolution analysis of dry samples. However, some amino acid fragments are excluded because they could not be resolved from PDMS interference peaks^21,22^. For example, CH_4_N^+^ (m/z 30), CH_3_N_2_^+^ (m/z 43), C_2_H_6_N^+^ (m/z 44), CHS^+^ (m/z 45), C_3_H_6_N^+^(m/z 56), C_2_H_7_N_3_^+^ (m/z 73) and C_9_H_7_O_2_^+^ (m/z 147) have the same m/z values with SiH_2_^+^, SiNH^+^, SiNH_2_^+^, SiNH_3_^+^, Si_2_^+^, Si(CH_3_)_3_^+^ and Si_2_(CH_3_)_5_O^+^ using unit mass, respectively.

The intensities of amino acid fragments also vary among these hydrated protein film samples, indicating the compositional difference of each amino acid in different protein molecules. As to the negative ion mode, because the hydrogen atoms are mostly lost, the amino acid fragments are rarely reported^9,10^. In addition, Fig. S5 depicts the ToF-SIMS spectra collected from different locations of the hydrated vitronectin film. These spectra have similarities in the spectra in the positive (Fig. S5a–c) and negative ion mode (Fig. S5d–f), respectively, implying a good uniformity of the hydrated protein film and showing measurement reproducibility.

## Discussion

Spectral principal component analysis (PCA) is conducted to understand of the molecular and structural differences among these hydrated protein films. Details of PCA are seen in the supplemental information (SI). To simplify the analysis, only amino acid fragments labelled red, water clusters labelled green (positive ion mode) in Fig. 2, and water clusters labelled blue (negative ion mode) in Fig. S1 are selected. These peaks are normalized to sum of the selected peak intensities, square root transformed, and mean cantered before performing the PCA.

**Figure 3:**
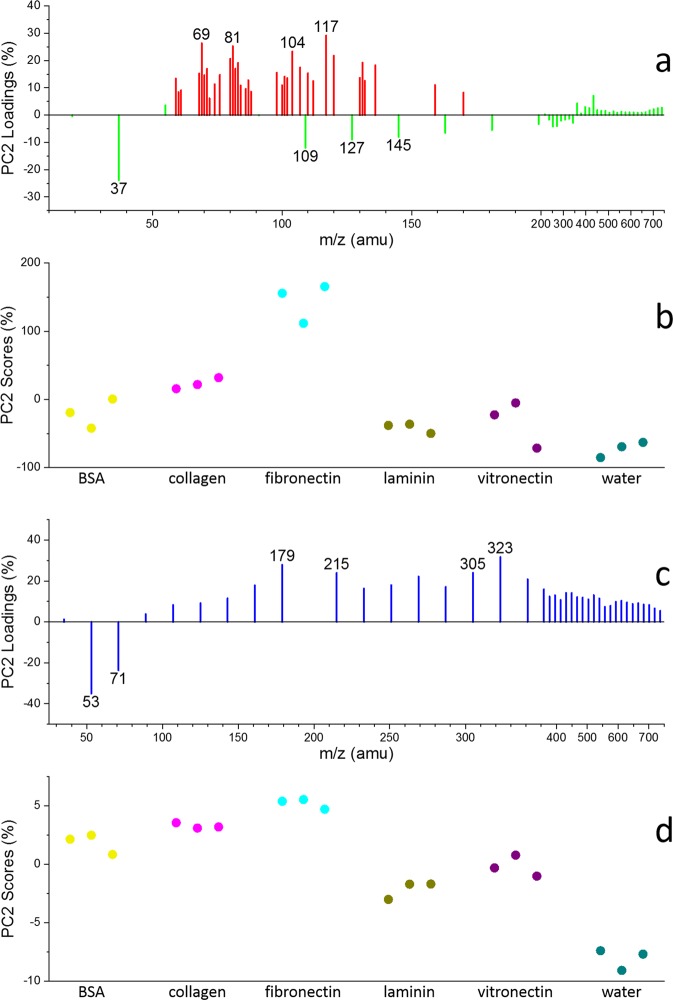
PCA results of six samples. Positive ion mode: (**a**) PC1 (69%) loadings of amino acid fragments (red) and water clusters (green) labelled in Fig. 2, (**b**) PC1 (69%) scores of six samples. Negative ion mode: (**c**) PC1 (76%) loadings of water clusters (blue) labelled in Fig. S1, (**d**) PC1 (76%) scores of six samples.

PC1 accounts for 69% of the total variance in the positive ion mode data set, the plots of PC1 (69%) loadings and scores (Fig. 3) are very interesting. Overall, the amino acid fragment peaks (*e.g*., C_4_H_5_O^+^ m/z 69, C_4_H_5_N_2_^+^ m/z 81, C_4_H_10_NS^+^ m/z 104, C_5_H_9_SO^+^ m/z 117) have high negative loadings, while the low mass (m/z < 350) water cluster peaks (*e.g*., (H_2_O)_2_H^+^ m/z 37, (H_2_O)_3_H^+^ m/z 55, (H_2_O)_5_H^+^ m/z 91, (H_2_O)_6_H^+^ m/z 109, (H_2_O)_7_H^+^ m/z 127, (H_2_O)_8_H^+^ m/z 145) have high positive loadings. Thus, the samples with lower PC1score values have higher amino acid contributions than others, while the samples with higher PC1 scores have higher water cluster content than others. Specifically, the PC1 score of each sample in Fig. 3b shows that the amino acid fragment (especially for threonine C_4_H_5_O^+^, histidine C_4_H_5_N_2_^+^, methionine C_4_H_10_NS^+^, C_5_H_9_SO^+^) contents of samples can be ranked as the following: fibronectin> collagen> BSA > vitronectin> laminin> water; while the water cluster components in these samples are sorted in a reverse order.

On the other hand, PC1 accounts for 76% of the total variance in the negative ion mode PCA results, and the PC1 loadings of water clusters (blue) are depicted in Fig. 3c. Excluding the influence of amino acid fragments, the PC1 loadings of water clusters in Fig. 3d are similar to those in Fig. 3c. The smaller water clusters (*e.g*., (H_2_O)_2_OH^−^ m/z 53, (H_2_O)_3_OH^−^ m/z 71) have positive values while the larger water clusters (*e.g*., (H_2_O)_11_OH^−^ m/z 215, (H_2_O)_16_OH^−^ m/z 305, (H_2_O)_17_ OH^−^ m/z 323) negative values, indicating that higher PC1 score samples should have higher content of smaller water clusters, and lower PC1 score samples higher content of larger water clusters. Smaller water clusters were reported to be more likely to form in hydrophilic materials, while larger water clusters in hydrophobic materials^25^. Our results show that the hydrophilicity/hydrophobicity of these hydrated protein films can be verified using *in situ* molecular imaging. Figure 3d depicts the PC1 score plot of these samples. The PC1 score can be ranked in the increasing order as the following: fibronectin <collagen <BSA < vitronectin <laminin <water. This ranking matches with the order in Fig. 3b. This result suggests the hydrophilicity/hydrophobicity order of these protein molecules.

It should be noted that in Fig. 3a the high mass water clusters share the same trend as that found in amino acid fragments, only low mass water clusters show an opposite trend as depicted. This finding is qualitatively consistent with results from those in the negative ion mode. This result could give more insights into the hydrophilicity/hydrophobicity of the corresponding protein molecules. When the protein molecule is more hydrophobic, it can bind fewer water molecules and its hydrated film would have more amino acid fragment content and less water cluster content. In addition, water molecules tend to form big clusters, like the formation of dew droplets on highly hydrophobic material surface as a lotus leave. When the protein molecule is more hydrophilic, it can bind more water molecules, its hydrated film would have more water cluster content and less amino acid fragment content. The water microenvironment is important in cell attachment and moderate cell membrane interactions with adjacent molecules in the liquid.

Additionally, selected peaks are normalized to H^+^ in the positive ion mode or H^−^ in the negative ion mode prior to performing additional PCA. More information is provided in the SI. In this PCA, PC1 accounts for 68% of the total variance in the positive ion mode data set, the PC1 loadings of amino acid fragments (red) and water clusters (green) are depicted in Fig. S6a, all of which have positive values. This may be caused by different film coverage and thickness of proteins^26^. We verify this postulation using quantitative x-ray photoelectron spectroscopy (XPS) (Fig. S7 and Table S3). The way the protein film prepared should produce monolayers as reported using ellipsometry under similar conditions previously^27^. Based on the PC1 scores of the samples in Fig. S6b, the total contents of amino acids and water clusters in the samples are sorted as: BSA > vitronectin> fibronectin> laminin> collagen> water. According to the working principles of liquid ToF-SIMS (Fig. 1a), the stiffness of the sample surface should have an influence on the number of secondary ions. The stiffer the sample is, the larger the total counts of secondary ions are. Thus, it is reasonable to predict the stiffness order of these hydrated protein films based on their normalized total intensities of amino acid fragments and water clusters, which might reflect the interactions among protein molecules in the film as soft materials.

In the negative ion mode data set, PC1 accounts for 91% of the total variance. Fig. S6c shows the PC1 loading of water clusters (blue), all have positive values. According to the PC1 scores of the samples in Fig. S6d, the total contents of water clusters are sorted as the following: BSA > vitronectin> laminin> water> fibronectin> collagen, which is also influenced by the stiffness of the materials^28^.

It should be noted that PC2 using normalization to the H^+^ or H^−^ intensities was extremely consistent with PC1 using normalization to sum of the selected peak intensities. This may be because the influence of total intensity difference on PCA should be considered. This result indicated that PC1 using normalization to the H^+^ or H^−^ intensities were related to the total intensity of samples. In other words, H^+^ or H^−^ intensities represent significant percentages in the total ion counts in this work.

Considering these PCA results together, in both positive and negative ion mode, the content of every amino acid fragment or water cluster in each hydrated protein film sample could be evaluated, as shown in Fig. S8. Thus, the key components such as water cluster and amino acid fragment composition of these hydrated protein films are semi-quantitatively determined. The conformational changes of the protein samples can be compared using a secondary ion ratio, indicating the strongest hydrophobic side chains to the total observed strongest hydrophilic amino acids^27^. The liquid SIMS comparison results are depicted in Fig. S9. The order of the ratios of the hydrophobic to hydrophilic fragments of the proteins is not the same as that derived from water clusters. The discrepancy may arise from exclusion of amino acid fragments with overlapping peaks that are nascent to the SAVLI cell.

Besides, the third round of PCA (only the amino acid fragments selected, normalized to the sum of the selected amino acid fragment intensities) was done to study the different pattern of amino fragments among hydrated protein films. The PCA results were shown in Fig. S16 and the representative amino acid intensities in Fig. S17, which indicated the different amino acid compositions of the samples.

Due to the surface sensitivity of ToF-SIMS, the outmost surface of the adsorbed protein films are measured^10^. The external few nm of the protein film is the most biologically relevant. The *in situ* liquid ToF-SIMS approach probes the top few nm of the liquid^29^. This was verified by an AFM measurement of the cell membrane^23^. Although our experiment only presents static protein films, it demonstrates the possibility to study dynamic protein interactions using this new approach to observe how proteins function in cell membranes and other important biointerfaces as soft matter. Because ToF-SIMS is a label free imaging mass spectrometry technique, it offers the chance to provide more molecular insight. The mass fragmentation pattern is an important factor in ToF-SIMS spectral analysis. Building a community reference library and employing other new approaches in big data analysis are needed for better interpretation of complex biological systems.

**Figure 4:**
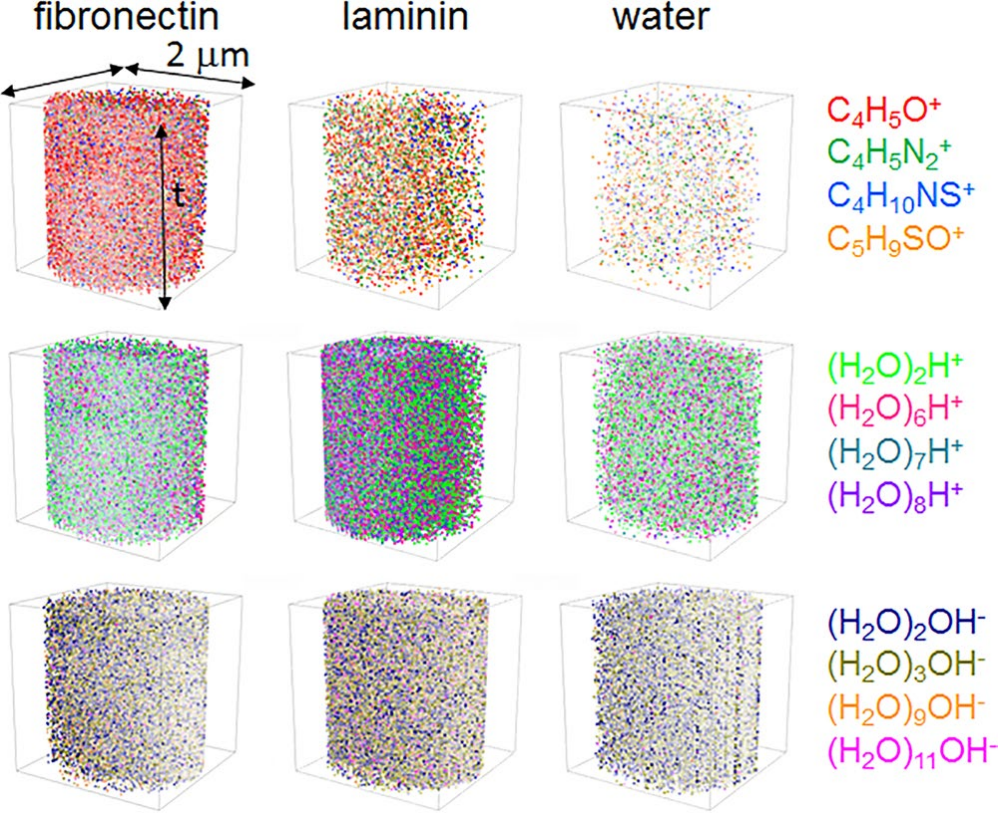
Normalized 3D images of selected positive amino acid fragments, positive water clusters and negative water clusters from hydrated fibronectin film, hydrated laminin film and water. Data were normalized to total ion counts and reconstructed from period II in Fig. 1c.

Furthermore, ToF-SIMS could provide 3D images of secondary ions as a molecular imaging technique. The visualized distribution of selected amino acids and water clusters in the hydrated protein film is shown in Fig. 4. The amino acids in hydrated fibronectin film are more than those in the hydrated laminin film, and even much more than those in water. Amino acid fragment signals in the water sample are mostly from the system background. While the total water cluster content in the hydrated fibronectin film is lower than those in hydrated laminin film and water, in the positive and negative ion mode. The lager water clusters in hydrated fibronectin and laminin films, such as (H_2_O)_6_H^+^, (H_2_O)_7_H^+^, (H_2_O)_8_H^+^ in the positive ion mode and (H_2_O)_9_OH^−^, (H_2_O)_11_OH^−^ in the negative ion mode, are more than those in DI water as expected. Our results intuitively presented the distribution difference of amino acids and water clusters of the relatively hydrophobic hydrated protein films (*e.g*., fibronectin), which have more similar composition to the dry protein film, and the relatively hydrophilic hydrated protein films (*e.g*., laminin), which have more similar composition to the water sample.

On potential advantage of *in situ* liquid SIMS to study protein films is that the protein structure is maintained in the *in situ* setup compared to the previous experiments done in UHV. Comparisons of the amino acid fragment patterns from a given protein analyzed in a dried state in UHV, a protected state (e.g., trehalose coated) in UHV^30,31^ and in the liquid cell of laminin and vitronectin were conducted. Spectral PCA results in Fig. S12 shows that the degree of protein denaturation would increase in the order of liquid state <protected UHV state <dried UHV state. This finding is supported by the increasing positive PC1 scores from the water clusters in the PCA analysis, indicative of the maintenance of the water clusters surrounding and enclosed within the protein monolayers.

In this study, several hydrated protein films were successfully immobilized in the SALVI microreactor and analysed by *in situ* liquid SIMS. The semi-quantitative observation of water clusters and amino acid fragments characterizes the detailed composition of water molecules and amino acid fragments of these hydrated monolayer protein films. The liquid ToF-SIMS spectral comparison and PCA results indicate the hydrophobicity and hydrophilicity difference of these hydrated protein films. The 3D images further demonstrate their spatial structures, which provides direct molecular visualization for molecular dynamic simulations of hydrated proteins. Thus, our work establishes an excellent platform for understanding the hydration mechanism of proteins and protein interactions at the solid-liquid interface in the future.

## Methods

### SALVI device fabrication

A schematic depicting the hydrated protein film adsorbed on the silicon nitride (SiN) membrane of SALVI is shown in Fig. 1a. The device was fabricated following the process described in previous papers^29,32–34^. Briefly, a PDMS block with a 200 μm wide, 300 μm deep and 2 mm long microchannel was made by soft lithography. A SiN membrane window consisting of a 7.5 × 7.5 mm^2^ silicon frame (200 μm thickness) and a 1.5 × 1.5 mm^2^ SiN membrane (100 nm thickness) acquired from Norcada, Canada was used to form the detection area. The SiN membrane and the PDMS microchannel were irreversibly bonded after oxygen plasma treatment.

### Hydrated protein film immobilization on the sin membrane

Five proteins including BSA (product number A4161), collagen (type IV, product number C8374), fibronectin (product number F1056), laminin (product number L6274) and vitronectin (product number V8379) were studied in this work, all purchased from Sigma-Aldrich, USA. The sterilization and cleaning of the SALVI microchannel was accomplished by washing it thoroughly with 1 mL of 70% ethanol solution and 1 mL of pure water (Milli-Q Advantage A10 Water Purification System, EMD Millipore, Germany), respectively, at a flow rate of 100 μL/min. Then the microchannel was filled with 100 μL of 10 μg/mL protein solution (neutral pH) at a flow rate of 100 μL/min and left incubated at room temperature for 12 hours^20^. Normally, a hydrated protein film would be immobilized in the microchannel^9,10,35–40^, as shown in Fig. 1a. The residual protein solution was removed by washing the channel with 1 mL of pure water at a flow rate of 100 μL/min. The trehalose preserved protein film samples were prepared following the same procedure described in earlier publications^30,31^.

### ToF-SIMS measurement

A TOF.SIMS 5 (IONTOF GmbH, Germany) was utilized in this study. The SALVI device was assembled on the ToF-SIMS stage before analysis, as shown in Fig. 1b. Specifically, a 25 keV Bi_3_^+^ primary ion beam with a spatial resolution of 400 nm and a beam current of 1.0 pA at a cycle time of 100 μs was used. As shown in Fig. 1c,a long pulse width (180 ns, in Region I, with higher intensity but lower mass resolution, *i.e*., ~100) was applied for punching through the SiN membrane. Once the detection area of 2 μm in diameter was formed^13,41^ and the SIMS depth profile became steady for about 100 s, a short pulse width (80 ns, with lower intensity but higher mass resolution, *i.e*., ~400) was applied for collecting spectrum data for at least 200 s in Region II. Raw data were reduced using the SurfaceLab 6 software (IONTOF GmbH, Germany). The SIMS data were mass calibrated using CH_3_^+^, C_2_H_5_^+^, C_3_H_7_^+^, and C_4_H_9_^+^ peaks for the positive ion mode spectra; and CH^−^, C_2_H^−^, C_3_H^−^, and C_4_H^−^ peaks for the negative ion mode spectra, respectively. As the reproducibility of this semi-quantitative technique was proven in Fig. S13 using nine replicates, three replicates for each sample was used. MATLAB R2105b (MathWorks, USA) was utilized for spectral PCA^9,10,36–40,42,43^. Peaks were selected for spectral PCA as shown in Tables S1 and S2. Prior to PCA, the m/z peaks were normalized to either the sum of the selected peak intensities or H^+^ counts in the positive ion mode and H^−^ counts in the negative ion mode, respectively. Data were then square-root transformed and meancentered before the PCA. Table S3 gives the confidence level limits for the PCA results reported in this paper^44^. PDMS peaks contribute to ~2% of the total ion intensity and not a major interference in analysis (Table S4).

### XPS experimental description

XPS data were acquired using a Physical Electronics Quantera Scanning X-ray Microprobe. This system uses a focused monochromatic Al Kα X-ray (1486.7 eV) source for excitation and a spherical section analyzer. The instrument has a 32 element multichannel detection system. The X-ray beam is incident normal to the sample and the photoelectron detector is at 45° off-normal. High energy resolution spectra were collected using a pass-energy of 69.0 eV with a step size of 0.125 eV. For the Ag 3d_5/2_ line, these conditions produced a FWHM of 0.92 eV ± 0.05 eV. The binding energy (BE) scale is calibrated using ISO 15472 Ed. 2 Surface Chemical Analysis - XPS - Calibration of energy scales. The Cu 2p_3/2_ feature is set at 932.62 ± 0.05 eV and Au 4f_7/2_ line was set at 83.96 ± 0.05 eV. Quantification was performed using Ulvac-phi Inc., MultiPak software version 9.1.1.7. Table S5 reports XPS analysis results.

## Supplementary information

Supplemental Information.

Supplemental Information 2

## Data Availability

All data generated or analyzed during this study are included in this published article (and its Supplementary Information files).
