# Supplementary material for: In situ molecular imaging of adsorbed protein films in water indicating hydrophobicity and hydrophilicity
Source: Sci Rep. 2020 Feb 28;10:3695. doi: 10.1038/s41598-020-60428-1 (PMC7048838; doi:10.1038/s41598-020-60428-1)
Supplement: Supplementary file 1 — Supplemental Information. [file 41598_2020_60428_MOESM1_ESM.pdf]

In situ molecular imaging of adsorbed protein films in water indicating hydrophobicity and hydrophilicity

Jiachao Yu,[abd] Yufan Zhou,[c] Mark Engelhard,[c] Yuchen Zhang,[b] Jiyoung Son,[b] Songqin Liu,[*a] Zihua Zhu,[*c] and Xiao-Ying Yu[*b]

[a] *Jiangsu Province Hi-Tech Key Laboratory for Bio-medical Research, School of Chemistry and Chemical Engineering, Southeast University, Nanjing 210096, China. E-mail: liusq@seu.edu.cn*

[b] *Energy and Environment Directorate, Pacific Northwest National Laboratory, Richland, WA 99354, USA. E-mail: xiaoying.yu@pnnl.gov*

[c] *Environmental Molecular Sciences Laboratory, Pacific Northwest National Laboratory, Richland, WA 99354, USA. E-mail: zihua.zhu@pnnl.gov*

[d] *Department of Chemistry, School of Science, Zhejiang Sci-Tech University, Hangzhou 310018, China.*

Table of Contents

EXPERIMENTAL DETAILS 1

SALVI Device Fabrication 1

Hydrated Protein Film Immobilization on the SiN Membrane 1

ToF-SIMS Measurement 1

XPS Experimental Description 2

SUPPLEMENTARY FIGURES 3

Fig. S1 ToF-SIMS spectra (m/z 1-200) of six samples: (a) BSA, (b) collagen, (c) fibronectin, (d) laminin, (e) vitronectin, (f) pure water in the negative ion mode. Water clusters were marked with blue labels. 3

Fig. S2 ToF-SIMS spectra (m/z 201-750) of six samples: (a) BSA, (b) collagen, (c) fibronectin, (d) laminin, (e) vitronectin, (f) pure water in the positive ion mode. Water clusters were marked with green labels. 4

Fig. S3 ToF-SIMS spectra (m/z 201-750) of six samples: (a) BSA, (b) collagen, (c) fibronectin, (d) laminin, (e) vitronectin, (f) pure water in the negative ion mode. Water clusters were marked with blue labels. 5

Fig. S4 ToF-SIMS spectra (m/z 1-200) of (a) hydrated BSA film and (b) dry BSA in the positive ion mode. Amino acid fragments were marked with red labels. 6

Fig. S5 ToF-SIMS spectra of hydrated vitronectin film at six different locations: (a), (b), (c) in the positive ion mode and (d), (e), (f) in the negative ion mode. Amino acid fragments were marked with red labels, water clusters were marked with green (in the positive ion mode) and blue (in the negative ion mode) labels. 7

Fig. S6 Spectral PCA results of six protein samples. (a) PC1 (68%) loading of amino acid fragments (red) and water clusters (green) depicted in Fig. 2 and S2, (b) PC1 (68%) score plot in the positive ion mode; (c) PC1 (91%) loading of water clusters (blue) depicted in Fig. S1 and S3, (d) PC1 (91%) score plot in the negative ion mode. 8

Fig. S7 Quantitative XPS analysis results of dry protein films. 9

Fig. S8 Comparison of selected (a) amino acid fragments, (b) positive water clusters and (c) negative water clusters in five hydrated protein films and pure water. 10

Fig. S9 Comparison bar plots showing the hydrophobic to hydrophilic amino acid ratios obtained from in situ liquid SIMS in this work. 11

Fig. S10 3D reconstructed images from a) absolute counts and b) relative counts normalized to the total ion intensities. 13

Fig. S11 3D reconstructed images of the laminin protein film: a) normalized to H+ or H- counts and b) normalized to the total ion intensities. 14

Fig. S12 PCA results of six samples. (a) PC1 (73%) loadings of amino acid fragments (red) and water clusters (green), (b) PC1 (73%) scores of six samples. 15

Fig. S13a ToF-SIMS spectra of 9 water replicates. Only water clusters were selected and showed, the intensities were normalized to sum of selected peak intensities. 16

Fig. S13b ToF-SIMS spectra of 9 laminin replicates. The intensities were normalized to the total ion intensity 17

Fig. S14a. Depth profiling measurement of the Laminin in the positive mode. 18

Fig. S14b. Depth profiling measurement of the Laminin with trehalose coating in the positive mode. 19

Fig. S15a. Depth profiling measurement of the Vitronectin in the positive mode. 20

Fig. S15b. Depth profiling measurement of the Vitronectin with trehalose coating in the positive mode. 21

SUPPLEMENTARY TABLES 24

Table S1 Water clusters observed in the positive and negative ion mode in this study 24

Table S2 Amino acid fragments observed in the positive ion mode in this study. 25

Table S3 95% confidence limits for all the PCA plots 26

Table S4 The relative abundance of PDMS in the liquid SIMS protein spectrum 27

Table S5 The XPS determined composition of BSA, laminin, and vitronectin films absorbed onto clean Si substrates 28

MOVIE CAPTION 29

REFERENCES 30

EXPERIMENTAL DETAILS

SALVI Device Fabrication

A schematic depicting the hydrated protein film adsorbed on the SiN membrane of SALVI is shown in Fig. 1a. The device was fabricated following the process described in previous papers.[1-4] Briefly, a polydimethylsiloxane (PDMS) block with a 200 μm wide, 300 μm deep and 2 mm long microchannel was made by soft lithography. A SiN membrane window consisting of a 7.5×7.5 mm^2 silicon frame (200 μm thickness) and a 1.5×1.5 mm^2 SiN membrane (100 nm thickness) acquired from Norcada, Canada was used to form the detection area. The SiN membrane and the PDMS microchannel were irreversibly bonded after oxygen plasma treatment.

Hydrated Protein Film Immobilization on the SiN Membrane

Five proteins including BSA (product number A4161), collagen (type IV, product number C8374), fibronectin (product number F1056), laminin (product number L6274) and vitronectin (product number V8379) were studied in this work, all purchased from Sigma-Aldrich, USA. The sterilization and cleaning of the SALVI microchannel was accomplished by washing it thoroughly with 1 mL of 70% ethanol solution and 1 mL of pure water (Milli-Q Advantage A10 Water Purification System, EMD Millipore, Germany), respectively, at a flow rate of 100 μL/min. Then the microchannel was filled with 100 μL of 10 μg/mL protein solution (neutral pH) at a flow rate of 100 μL/min and left incubated at room temperature for 12 hours.[5] Normally, a hydrated protein film would be immobilized in the microchannel,[6-13] as shown in Fig. 1a. The residual protein solution was removed by washing the channel with 1 mL of pure water at a flow rate of 100 μL/min.

ToF-SIMS Measurement

A TOF.SIMS 5 (IONTOF GmbH, Germany) was utilized in this study. The SALVI device was assembled on the ToF-SIMS stage before analysis, as shown in Fig. 1b. Specifically, a 25 keV Bi_3^+ primary ion beam with a spatial resolution of 400 nm and a beam current of 1.0 pA at a cycle time of 100 μs was used. As shown in Fig. 1c, a long pulse width (180 ns, in Region I, with higher intensity but lower mass resolution, *i.e.*, ~100) was applied for punching through the SiN membrane. Once the detection area of 2 μm in diameter was formed[14, 15] and the SIMS depth profile became steady for about 100 s, a short pulse width (80 ns, with lower intensity but higher mass resolution, *i.e.*, ~400) was applied for collecting spectrum data for at least 200 s in Region II. Raw data were reduced using the SurfaceLab 6 software (IONTOF GmbH, Germany). The SIMS data were mass calibrated using CH_3^+, $C_2H_5^+$, $C_3H_7^+$, $C_4H_9^+$ peaks for the positive ion mode spectra; and CH^-, C_2H^-, C_3H^-, C_4H^- peaks for the negative ion mode spectra. MATLAB R2105b (MathWorks, USA) was utilized for spectral principal component analysis (PCA).[7-13, 16, 17] Specific peaks were selected for spectral PCA, as shown in Table S1 and S2. Prior to PCA, the m/z peaks were normalized to H^+ counts in the positive ion mode and H^- counts in the negative ion mode, respectively. Data were then square-root transformed and meancentered before the PCA.

XPS Experimental Description

XPS data were acquired using a Physical Electronics Quantera Scanning X-ray Microprobe. A focused monochromatic Al Ka X-ray (1486.7 eV) excitation source and a spherical section analyzer were used in this system. The instrument had a multi-channel inspection system capable of detection of 32 elements. The X-ray beam was perpendicular to the sample and the photoelectron detector was offset from the normal by 45°. By using a pass-energy of 69.0 eV, high energy resolution spectra were collected in steps of 0.125 eV. A FWHM of 0.92 eV ± 0.05 eV was induced by the Ag 3d5/2 line. Calibration of the binding energy (BE) scale was conducted using ISO 15472 Ed. 2 Surface Chemical Analysis - XPS - Calibration of energy scales. The Cu 2p3/2 feature and Au 4f7/2 line were set at 932.62 ± 0.05 eV and 83.96 ± 0.05 eV, respectively. Quantification was performed using the Multi Pak software version 9.1.1.7 (Ulvac-phi Inc.).

SUPPLEMENTARY FIGURES

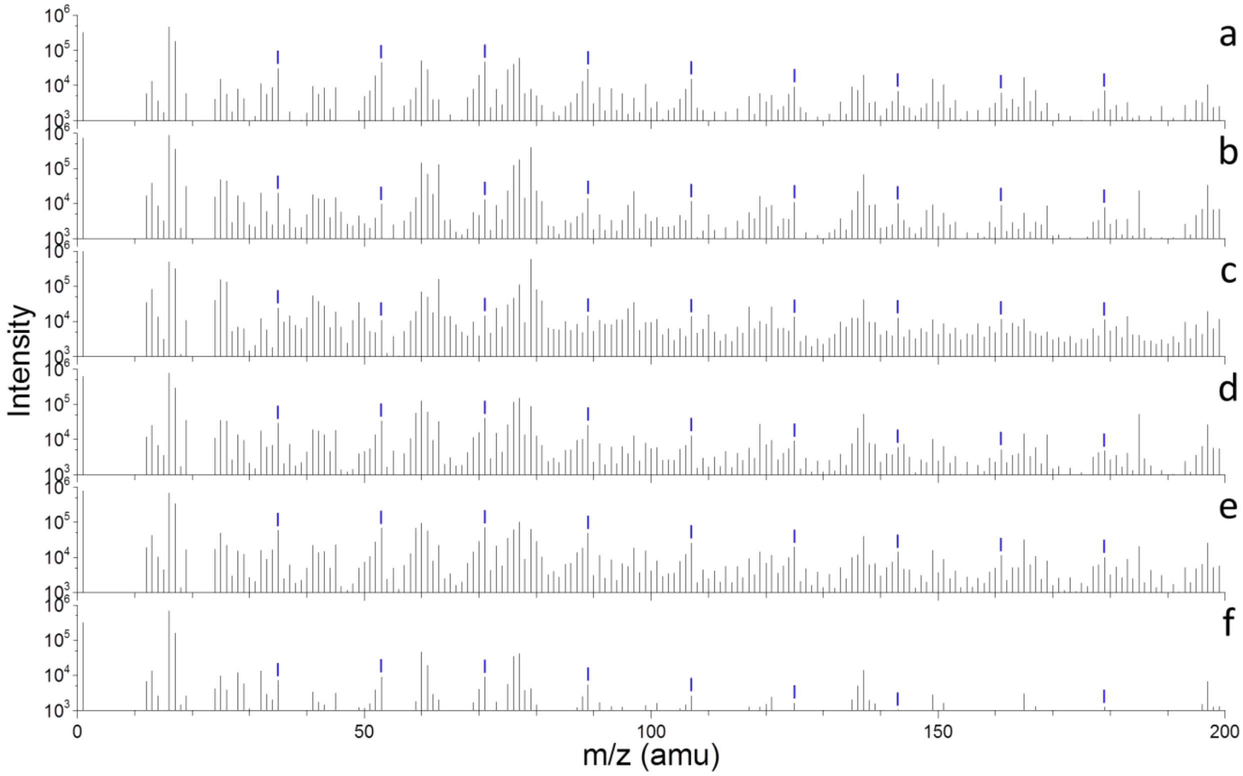

Fig. S1 ToF-SIMS spectra (m/z 1-200) of six samples: (a) BSA, (b) collagen, (c) fibronectin, (d) laminin, (e) vitronectin, (f) pure water in the negative ion mode. Water clusters were marked with blue labels.

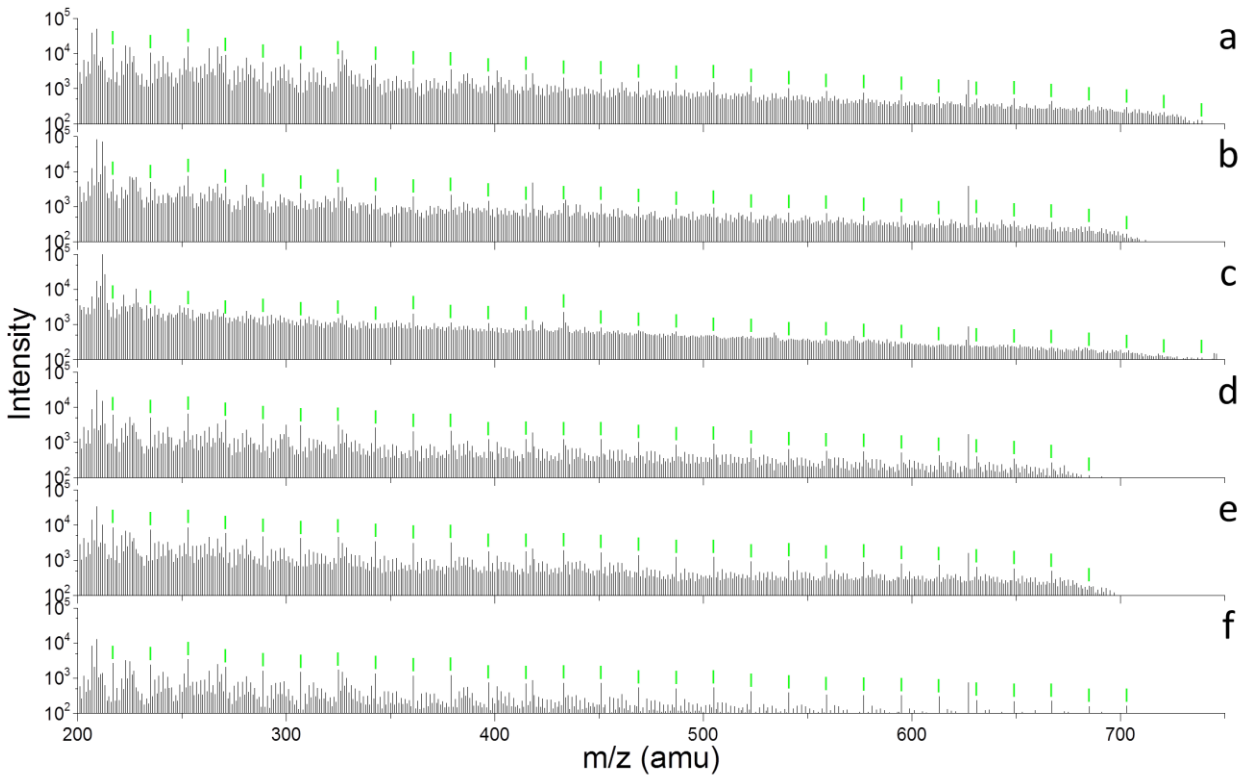

Fig. S2 ToF-SIMS spectra (m/z 201-750) of six samples: (a) BSA, (b) collagen, (c) fibronectin, (d) laminin, (e) vitronectin, (f) pure water in the positive ion mode. Water clusters were marked with green labels.

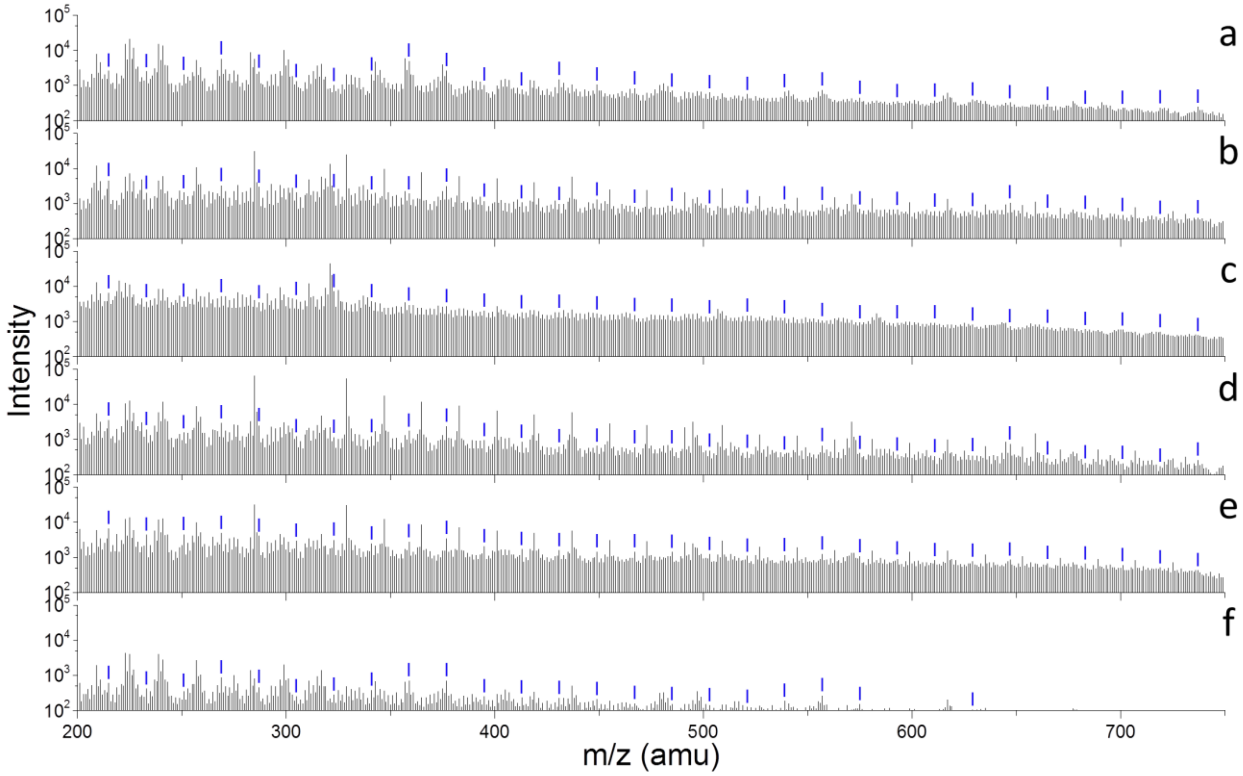

Fig. S3 ToF-SIMS spectra (m/z 201-750) of six samples: (a) BSA, (b) collagen, (c) fibronectin, (d) laminin, (e) vitronectin, (f) pure water in the negative ion mode. Water clusters were marked with blue labels.

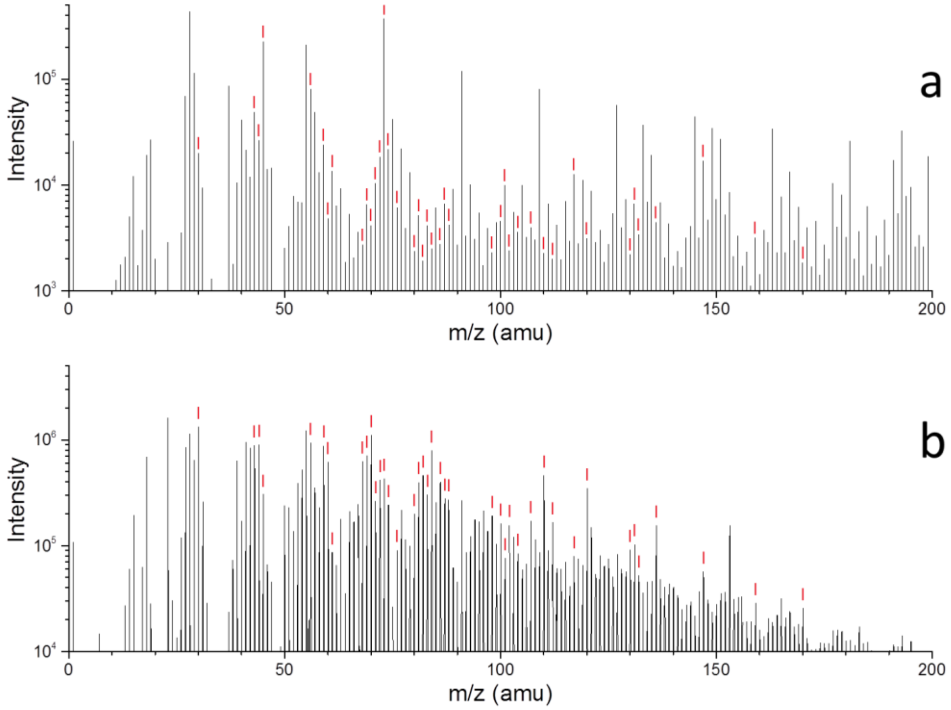

Fig. S4 ToF-SIMS spectra (m/z 1-200) of (a) hydrated BSA film and (b) dry BSA in the positive ion mode. Amino acid fragments were marked with red labels.

The hydrated BSA sample was analyzed using liquid SIMS with high spatial resolution and the dry BSA was analyzed using high mass resolution (*i.e.*, ~12000). This comparison shows that key peaks of amino acid fragments were observed in the hydrated and dry BSA samples, although observations of water clusters were not possible using the dry protein samples because of the nature of the sample preparation process.

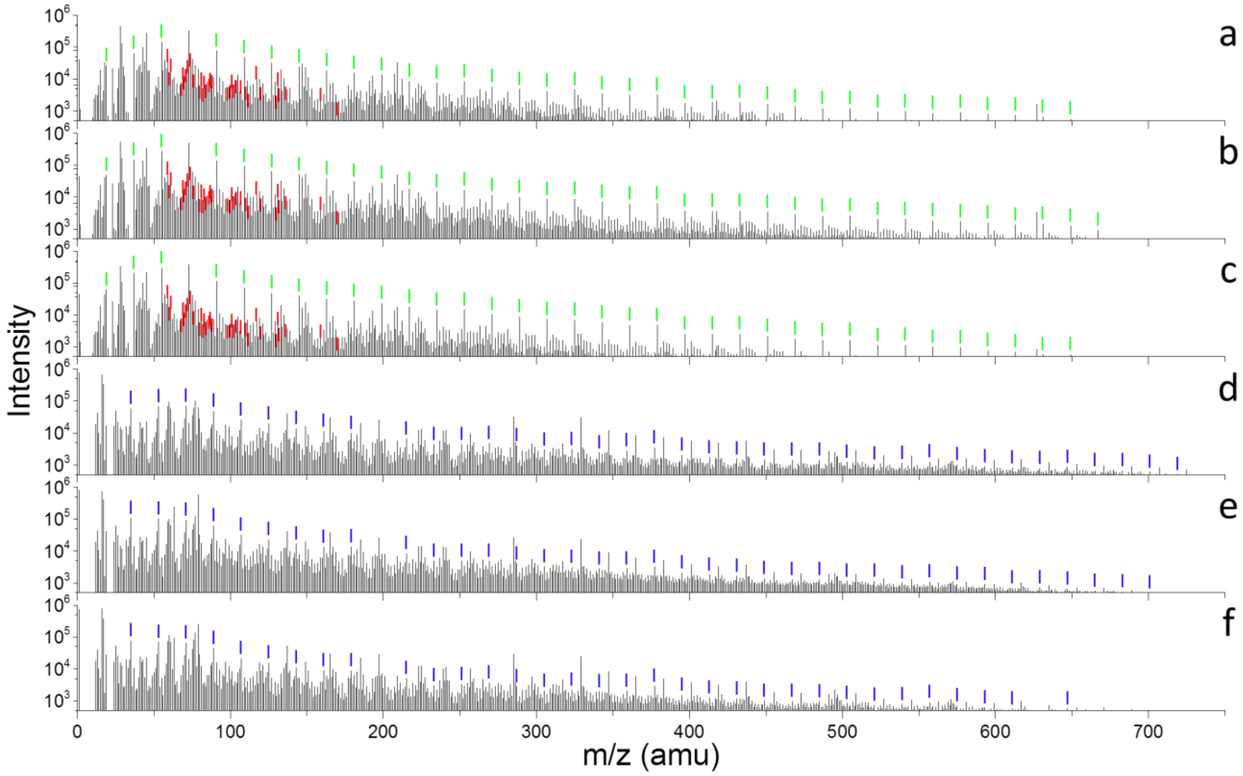

Fig. S5 ToF-SIMS spectra of hydrated vitronectin film at six different locations: (a), (b), (c) in the positive ion mode and (d), (e), (f) in the negative ion mode. Amino acid fragments were marked with red labels, water clusters were marked with green (in the positive ion mode) and blue (in the negative ion mode) labels.

Data acquired at different locations along the microfluidic channel showed that the reproducibility was reasonable among sampling spots. The counts of water clusters and amino acid fragments from each analysis were comparable.

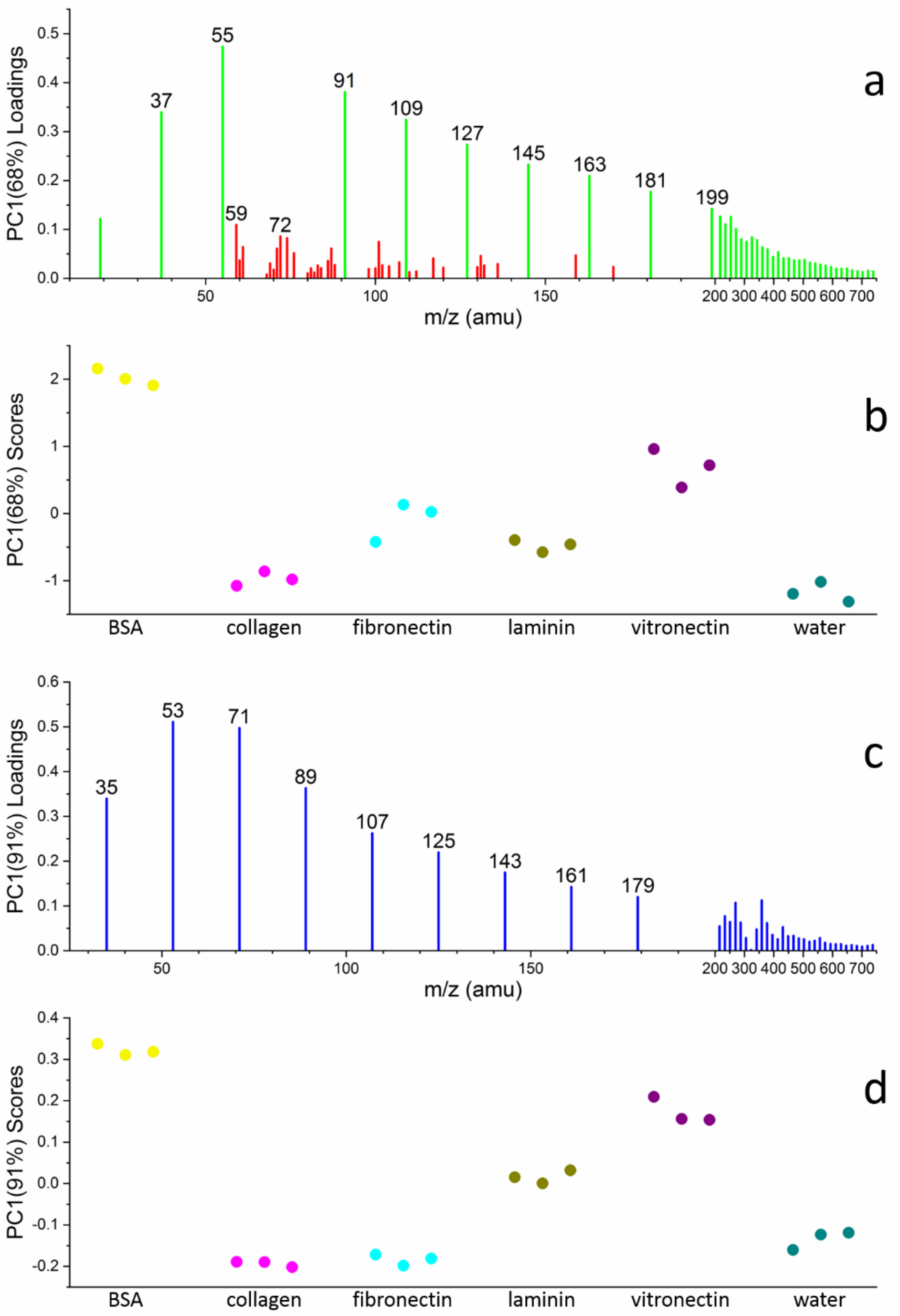

Fig. S6 Spectral PCA results of six protein samples. (a) PC1 (68%) loading of amino acid fragments (red) and water clusters (green) depicted in Fig. 2 and S2, (b) PC1 (68%) score plot in the positive ion mode; (c) PC1 (91%) loading of water clusters (blue) depicted in Fig. S1 and S3, (d) PC1 (91%) score plot in the negative ion mode.

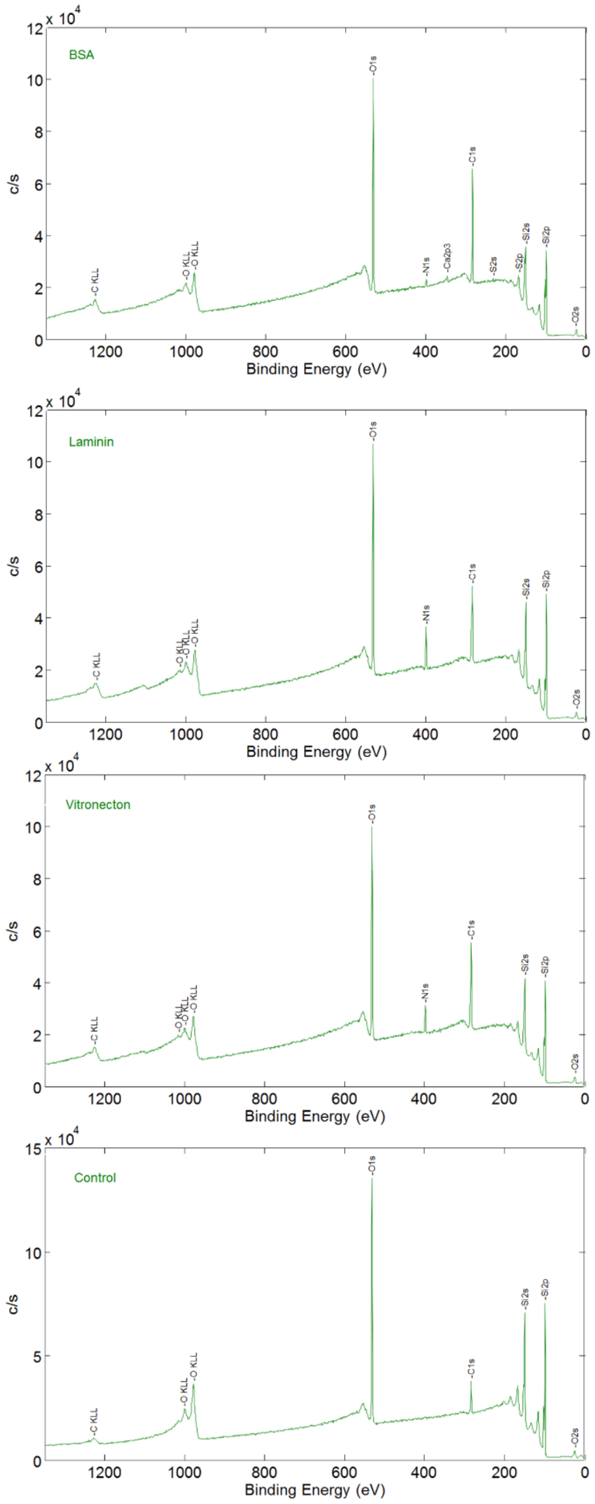

Fig. S7 Quantitative XPS analysis results of dry protein films.

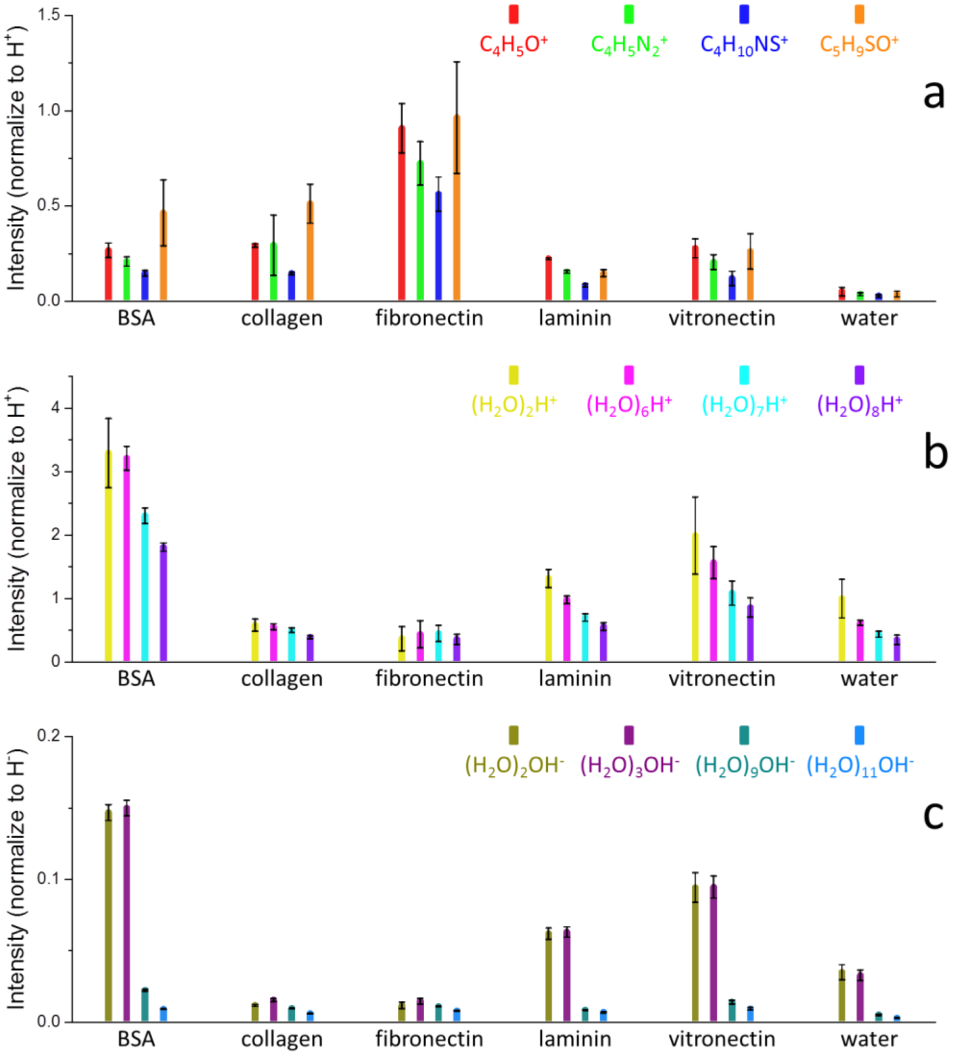

Fig. S8 Comparison of selected (a) amino acid fragments, (b) positive water clusters and (c) negative water clusters in five hydrated protein films and pure water.

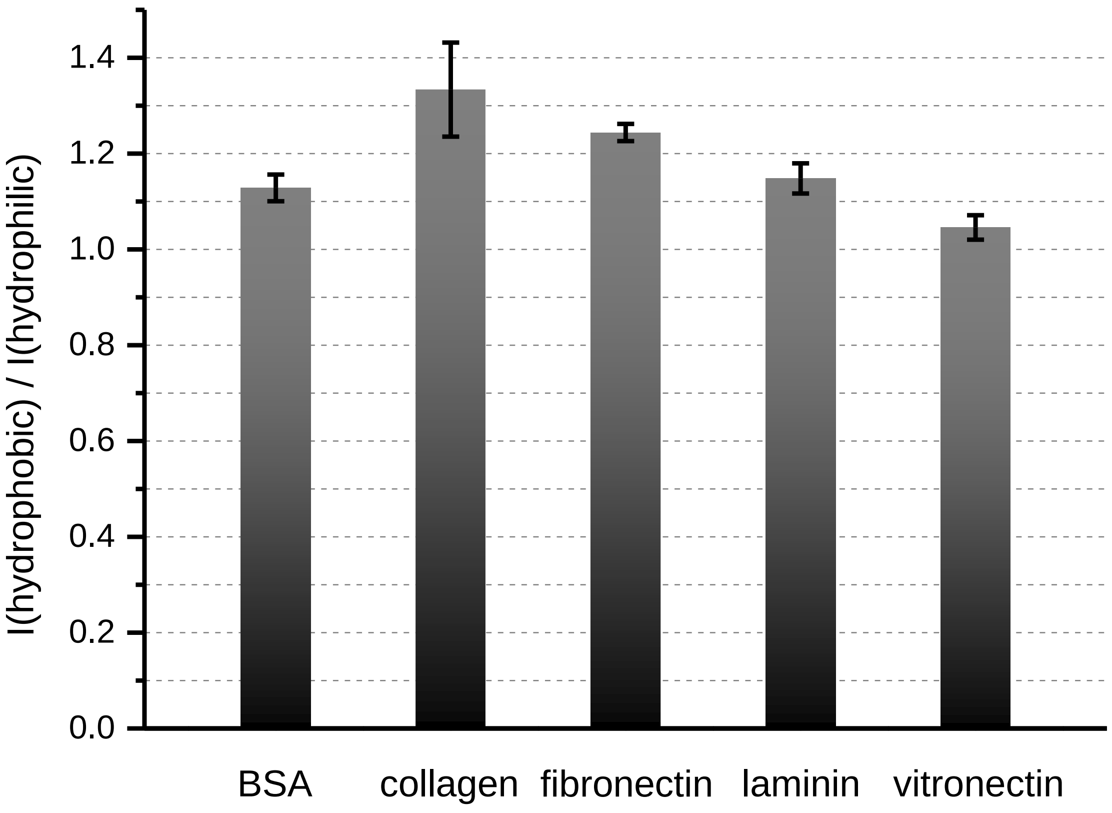

Fig. S9 Comparison bar plots showing the hydrophobic to hydrophilic amino acid ratios obtained from in situ liquid SIMS in this work.

The ratio of hydrophobic to hydrophilic amino acids follows the same method in J. Baio's paper in 2011.[18] Seven strongest hydrophobic amino acids (6 amino acids and 12 amino acid fragments selected) including the following:

isoleucine [Ile]: $C_5H_{12}N^+$ (isomer of leucine, same amino acid fragment, not shown in Table S2).

phenylalanine [Phe]: $C_8H_{10}N^+$, $C_9H_7O^+$, $C_9H_8O^+$

leucine [Leu]: $C_5H_{12}N^+$

methionine [Met]: $C_2H_5S^+$, $C_4H_{10}NS^+$, $C_5H_9SO^+$

proline [Pro]: $C_4H_6N^+$, $C_4H_8N^+$, $C_5H_6N^+$

valine [Val]: $C_4H_{10}N^+$, $C_5H_7O^+$.

Alanine [Ala]: $C_2H_6N^+$ is not included because of concurrence of $SiNH_2^+$.

6 strongest hydrophilic amino acids (12 amino acid fragments selected):

aspartic acid [Asp]: $C_3H_6NO_2^+$

arginine [Arg]: $CH_5N_3^+$, $C_4H_{10}N_3^+$, $C_4H_{11}N_3^+$, $C_5H_{10}N_3^+$

glutamic acid [Glu]: $C_4H_8NO_2^+$

lysine [Lys]: $C_5H_{10}N^+$

histidine [His]: $C_4H_5N_2^+$, $C_4H_6N_2^+$, $C_5H_8N_3^+$

asparagine [Asn]: $C_3H_7N_2O^+$, $C_4H_4NO_2^+$

During peak selection, $CH_3N_2^+$ is not included due to interference with $SiNH^+$, $C_2H_7N_3^+$ is excluded due to interference with $Si(CH_3)_3^+$ as relevant arginine peaks. The peak of $C_3H_6N^+$ interfered by Si_2^+ is not selected due to possible interference for lysine.

The ratio calculation results are not in excellent agreement with those from PCA, the reason might be that some amino acid fragments were excluded due to potential interference.

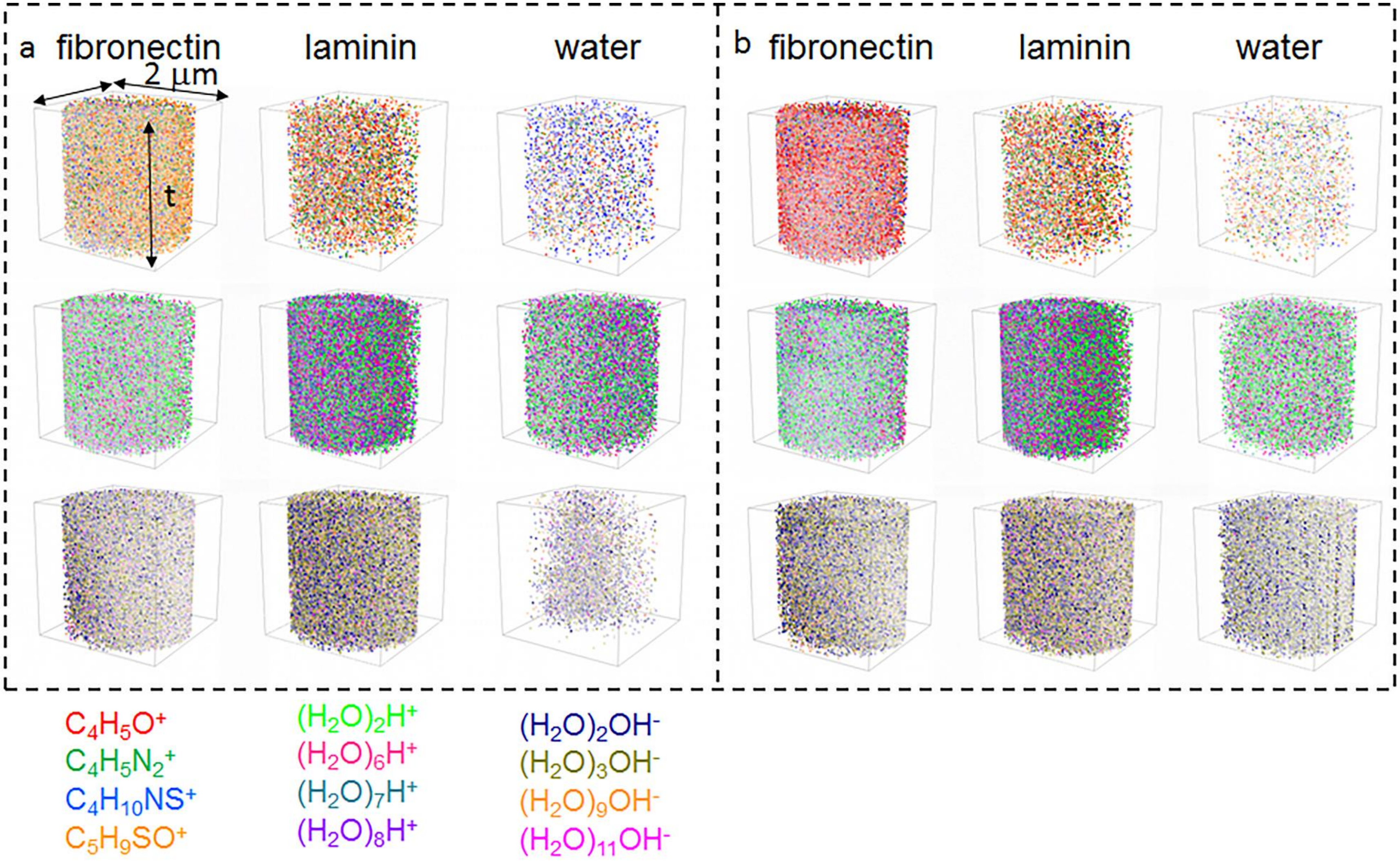

Fig. S10 3D reconstructed images from a) absolute counts and b) relative counts normalized to the total ion intensities. The comparisons show that the overall distributions do not vary too much; some ions like water clusters have improved visualization after normalization. Normalization makes more sense for comparison of liquid SIMS data.

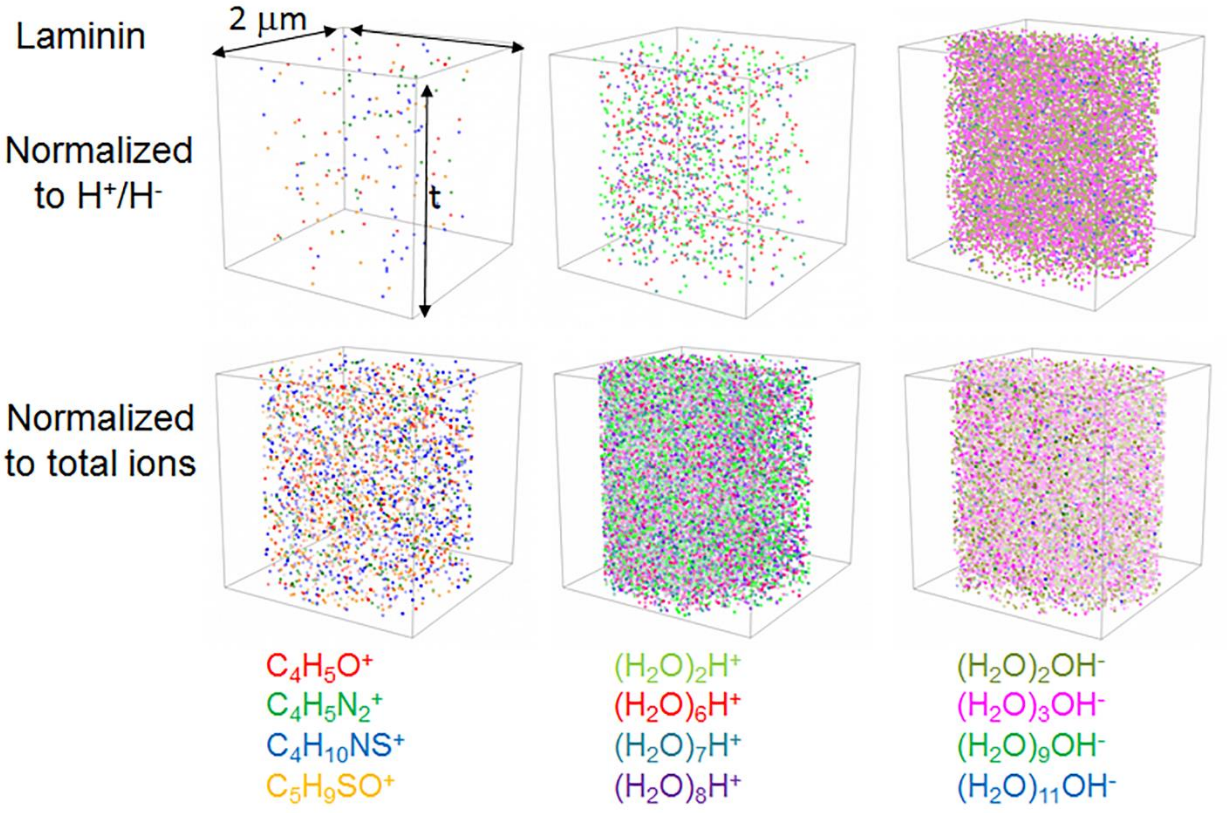

Fig. S11 3D reconstructed images of the laminin protein film: a) normalized to H^+ or H^- counts and b) normalized to the total ion intensities.

The comparisons show that normalization to H^+ or H^- does not necessarily give the best presentation of amino acid fragments and some of the water clusters. Normalization to the total ions gives better results.

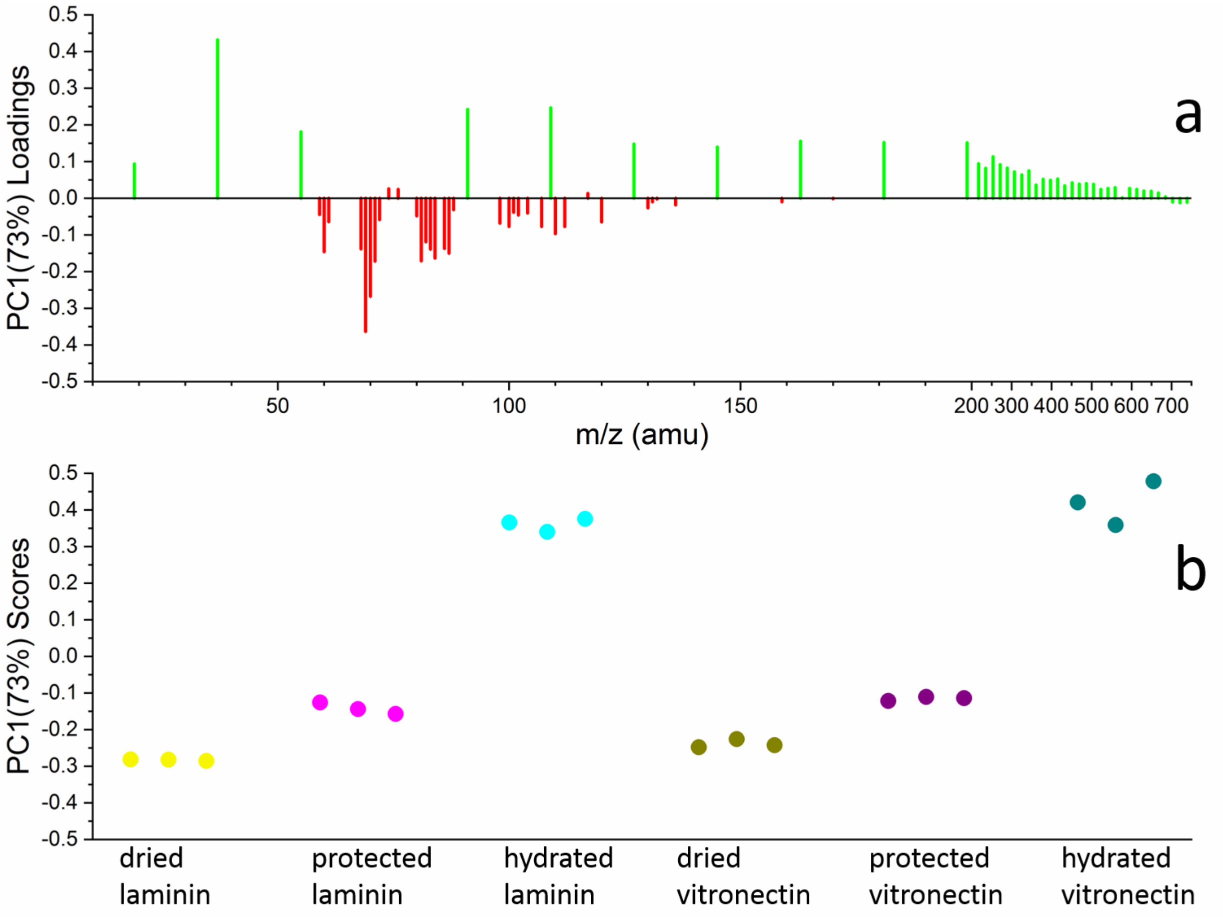

Fig. S12 PCA results of six samples. (a) PC1 (73%) loadings of amino acid fragments (red) and water clusters (green), (b) PC1 (73%) scores of six samples.

For both laminin and vitronectin, the scores were in the following order: dried protein film < trehalose protected protein film < hydrated protein film. Such result indicated that the degree of protein denaturation would decrease in the order: dried protein film > trehalose protected protein> hydrated protein.

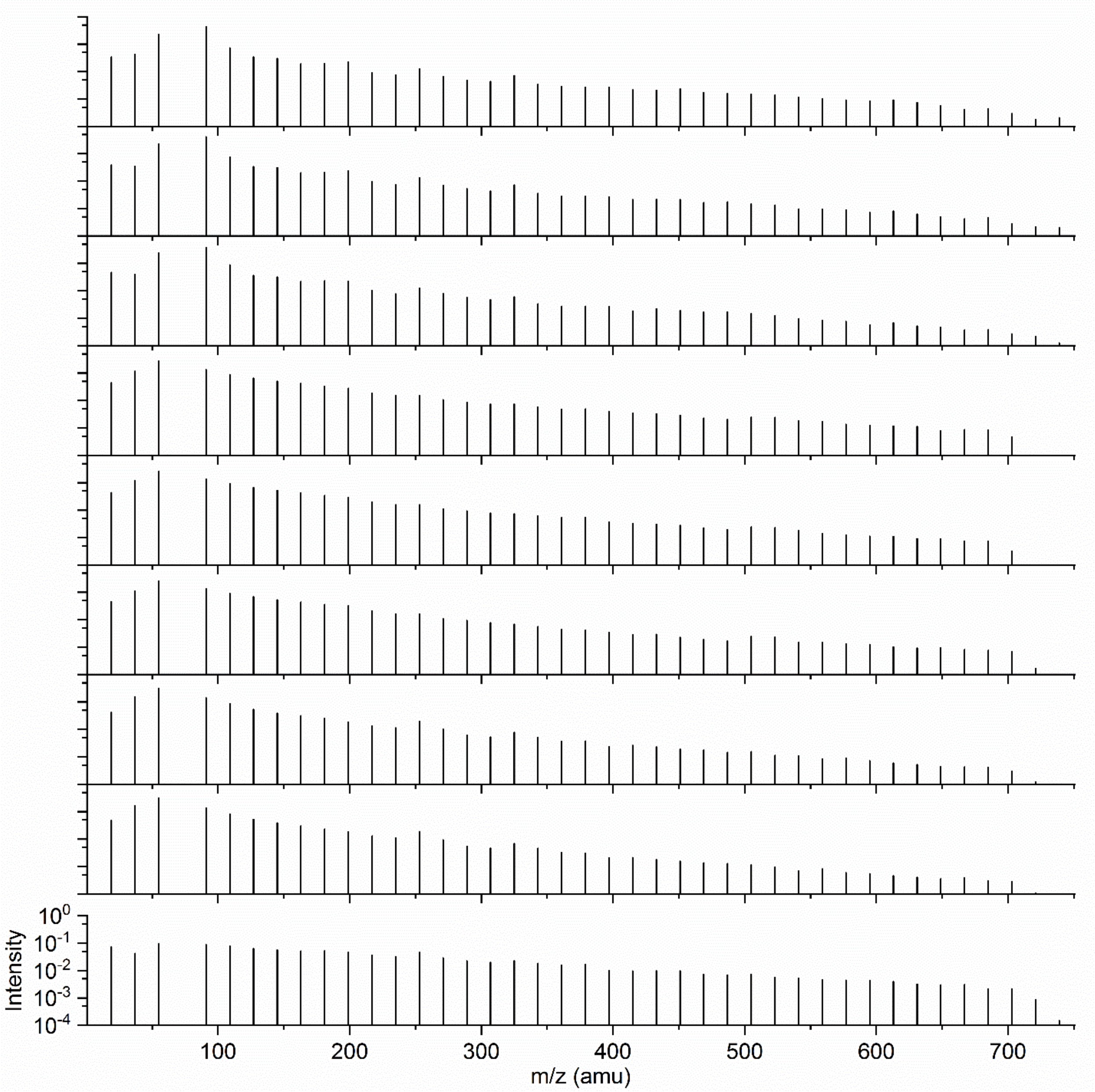

Fig. S13a ToF-SIMS spectra of 9 water replicates. Only water clusters were selected and showed, the intensities were normalized to sum of selected peak intensities.

There is no obvious difference among the replicates as a semi-quantitative technique, and the measurement reproducibility was illustrated. In the main text, fewer replicates for each sample were used.

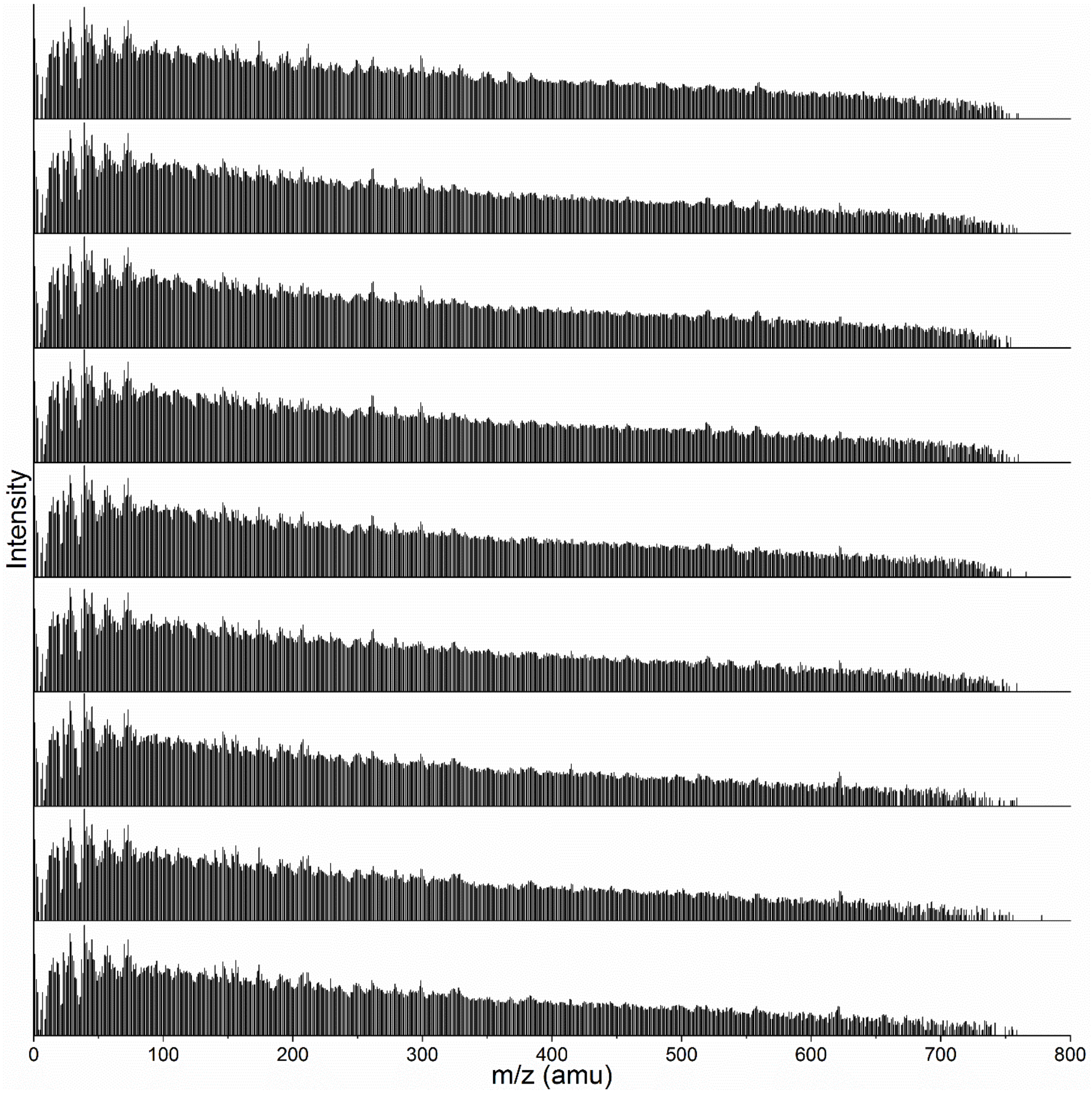

Fig. S13b ToF-SIMS spectra of 9 laminin replicates. The intensities were normalized to the total ion intensity.

There is no obvious difference among the replicates using in situ liquid SIMS, and the measurement reproducibility was illustrated. In the main text, fewer replicates for each sample were used.

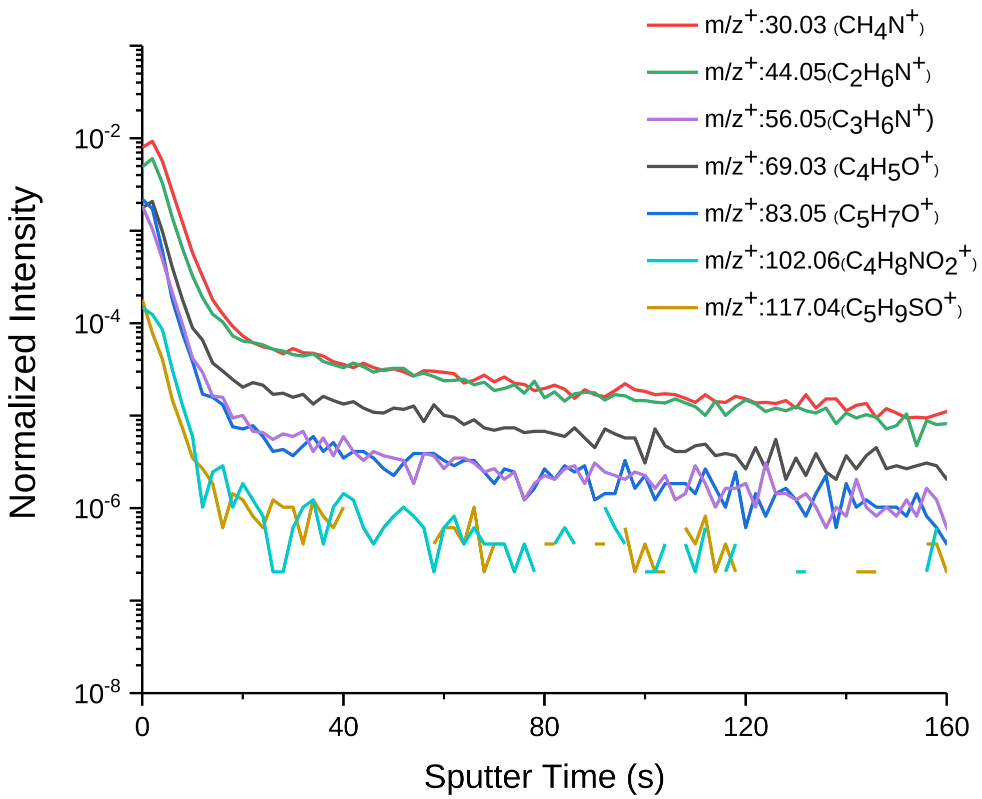

Fig. S14a. Depth profiling measurement of the Laminin in the positive mode.

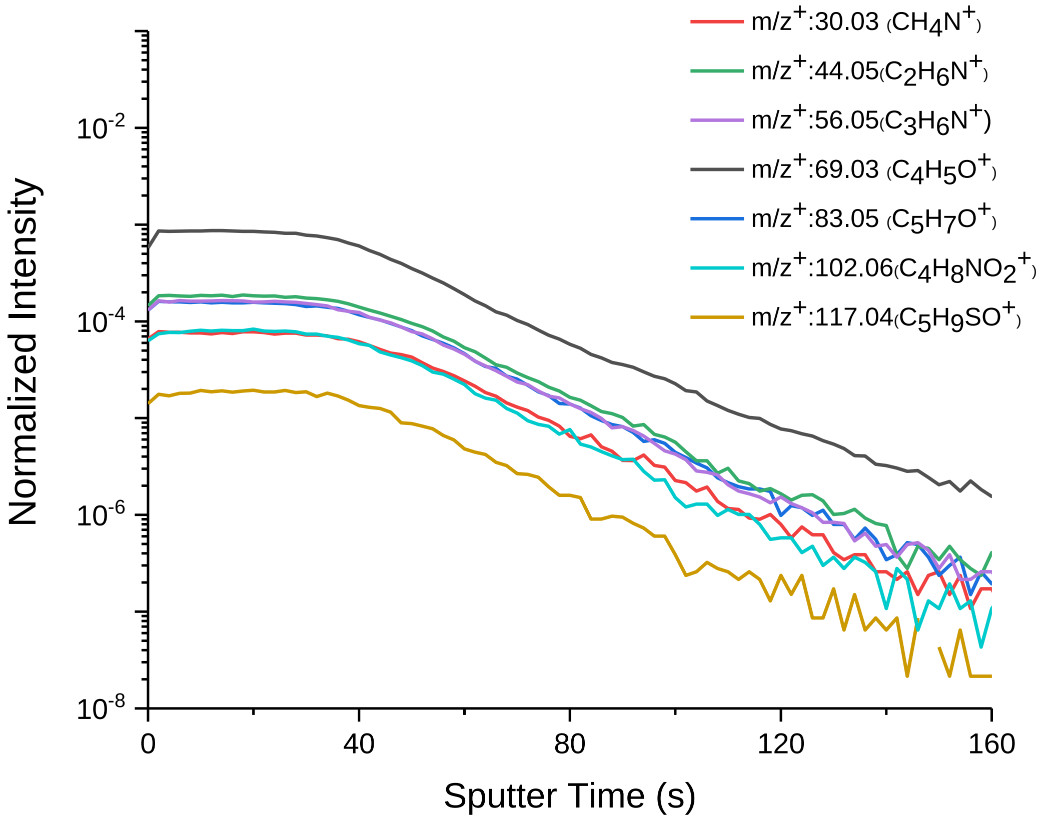

Fig. S14b. Depth profiling measurement of the Laminin with trehalose coating in the positive mode.

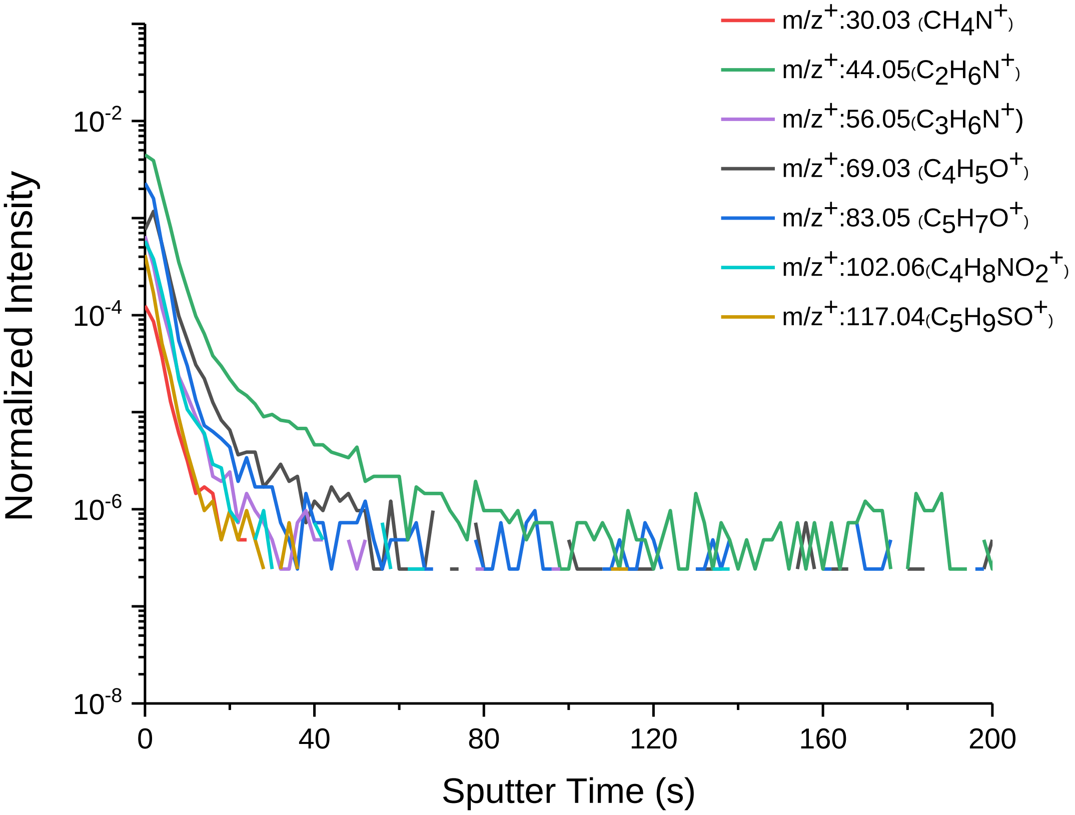

Fig. S15a. Depth profiling measurement of the Vitronectin in the positive mode.

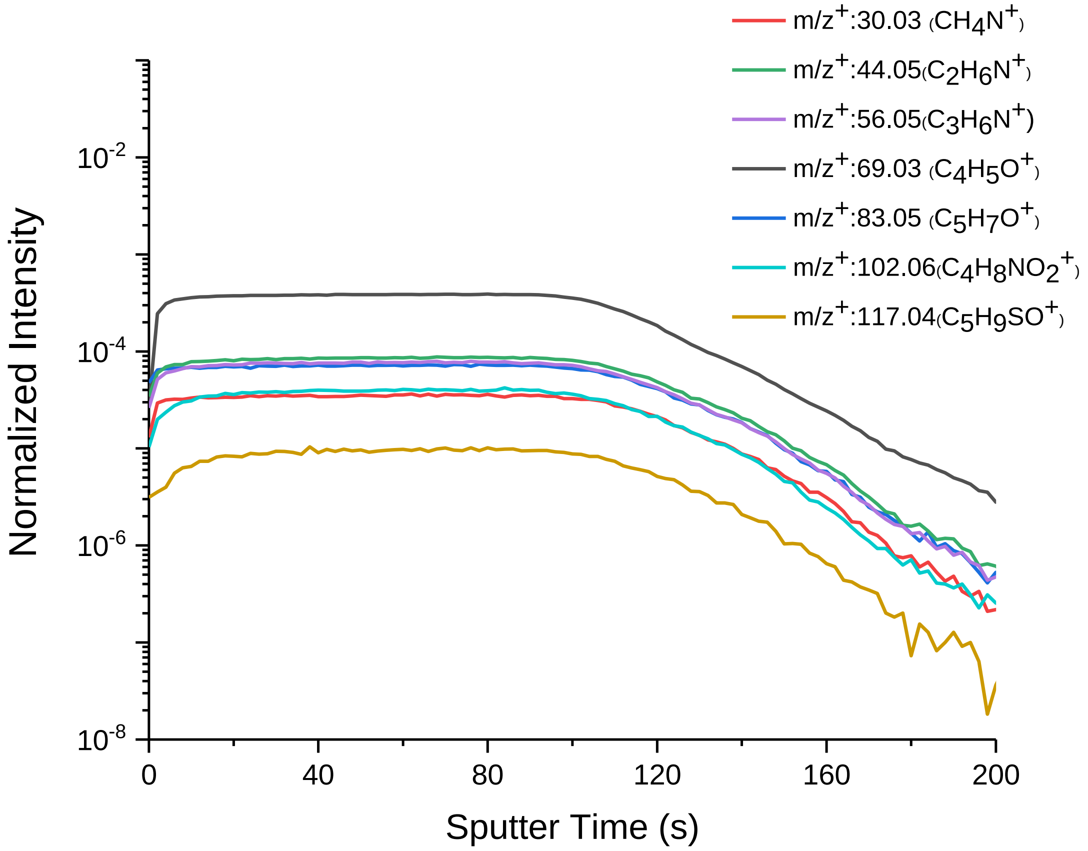

Fig. S15b. Depth profiling measurement of the Vitronectin with trehalose coating in the positive mode.

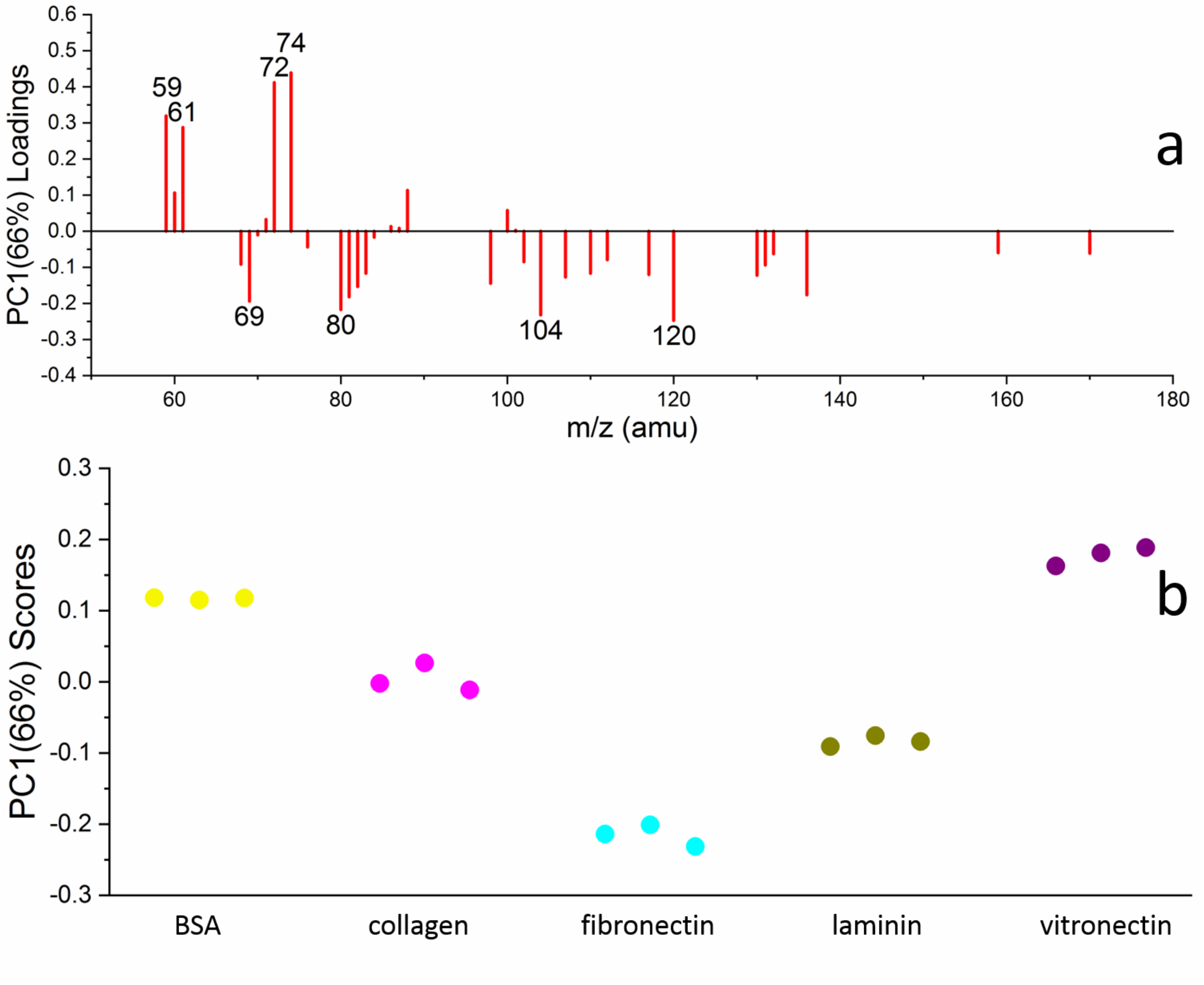

Fig. S16 PCA results of five samples. (a) PC1 (66%) loadings of amino acid fragments, (b) PC1 (66%) scores of five samples.

The amino acid fragment peaks (set 1, e.g., $CH_5N_3^+$ m/z 59, $C_2H_5S^+$ m/z 61, $C_4H_{10}N^+$ m/z 72, $C_3H_8NO^+$ m/z 74) have high positive loadings, while the amino acid fragment peaks (set 2, e.g., $C_4H_5O^+$ m/z 69, $C_5H_6N^+$ m/z 80, $C_4H_{10}NS^+$ m/z 104, $C_8H_{10}N^+$ m/z 120) have high negative loadings.

The samples with higher PC1 (69%) scores would have higher content of set 1 amino acids, while the samples with lower PC1 (69%) scores would have higher content of set 2 amino acids.

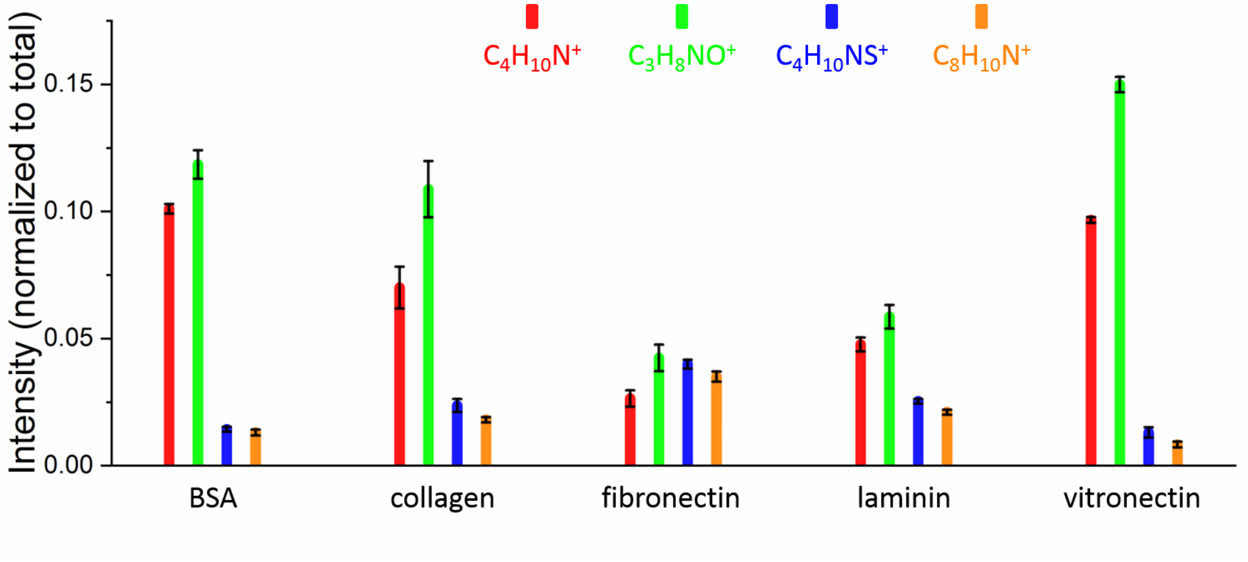

Fig. S17 Comparison of selected amino acid fragments (normalized to sum of the selected amino acid fragment intensities) in five hydrated protein films.

Four representative amino acids were selected to show the different amino acid compositions between samples. The results were consistent with Fig. S16.

SUPPLEMENTARY TABLES

Table S1 Water clusters observed in the positive and negative ion mode in this study.

positive ion mode			negative ion mode		
water cluster	m/z	interference	water cluster	m/z	interference
$(H_2O)H^+$	19		$(H_2O)OH^-$	35	
$(H_2O)_2H^+$	37		$(H_2O)_2OH^-$	53	
$(H_2O)_3H^+$	55		$(H_2O)_3OH^-$	71	
$(H_2O)_4H^+$	73	$SiC_3H_9^+$	$(H_2O)_4OH^-$	89	
$(H_2O)_5H^+$	91		$(H_2O)_5OH^-$	107	
$(H_2O)_6H^+$	109		$(H_2O)_6OH^-$	125	
$(H_2O)_7H^+$	127		$(H_2O)_7OH^-$	143	
$(H_2O)_8H^+$	145		$(H_2O)_8OH^-$	161	
$(H_2O)_9H^+$	163		$(H_2O)_9OH^-$	179	
$(H_2O)_{10}H^+$	181		$(H_2O)_{10}OH^-$	197	$(SiO_2)_3OH^-$
$(H_2O)_{11}H^+$	199		$(H_2O)_{11}OH^-$	215	
$(H_2O)_{12}H^+$	217		$(H_2O)_{12}OH^-$	233	
$(H_2O)_{13}H^+$	235		$(H_2O)_{13}OH^-$	251	
$(H_2O)_{14}H^+$	253		$(H_2O)_{14}OH^-$	269	
$(H_2O)_{15}H^+$	271		$(H_2O)_{15}OH^-$	287	
$(H_2O)_{16}H^+$	289		$(H_2O)_{16}OH^-$	305	
$(H_2O)_{17}H^+$	307		$(H_2O)_{17}OH^-$	323	
$(H_2O)_{18}H^+$	325		$(H_2O)_{18}OH^-$	341	
$(H_2O)_{19}H^+$	343		$(H_2O)_{19}OH^-$	359	
$(H_2O)_{20}H^+$	361		$(H_2O)_{20}OH^-$	377	
$(H_2O)_{21}H^+$	379		$(H_2O)_{21}OH^-$	395	
$(H_2O)_{22}H^+$	397		$(H_2O)_{22}OH^-$	413	
$(H_2O)_{23}H^+$	415		$(H_2O)_{23}OH^-$	431	
$(H_2O)_{24}H^+$	433		$(H_2O)_{24}OH^-$	449	
$(H_2O)_{25}H^+$	451		$(H_2O)_{25}OH^-$	467	
$(H_2O)_{26}H^+$	469		$(H_2O)_{26}OH^-$	485	
$(H_2O)_{27}H^+$	487		$(H_2O)_{27}OH^-$	503	
$(H_2O)_{28}H^+$	505		$(H_2O)_{28}OH^-$	521	
$(H_2O)_{29}H^+$	523		$(H_2O)_{29}OH^-$	539	
$(H_2O)_{30}H^+$	541		$(H_2O)_{30}OH^-$	557	
$(H_2O)_{31}H^+$	559		$(H_2O)_{31}OH^-$	575	
$(H_2O)_{32}H^+$	577		$(H_2O)_{32}OH^-$	593	
$(H_2O)_{33}H^+$	595		$(H_2O)_{33}OH^-$	611	
$(H_2O)_{34}H^+$	613		$(H_2O)_{34}OH^-$	629	
$(H_2O)_{35}H^+$	631		$(H_2O)_{35}OH^-$	647	
$(H_2O)_{36}H^+$	649		$(H_2O)_{36}OH^-$	665	
$(H_2O)_{37}H^+$	667		$(H_2O)_{37}OH^-$	683	
$(H_2O)_{38}H^+$	685		$(H_2O)_{38}OH^-$	701	
$(H_2O)_{39}H^+$	703		$(H_2O)_{39}OH^-$	719	
$(H_2O)_{40}H^+$	721		$(H_2O)_{40}OH^-$	737	
$(H_2O)_{41}H^+$	739				

Table S2 Amino acid fragments observed in the positive ion mode in this study.

amino acid fragment	amino acid	exact m/z	m/z (dry)	m/z (hydrated)	interference
CH_4N^+	glycine	30.03	30.03	30	SiH_2^+
$CH_3N_2^+$	arginine	43.03	43.03	43	$SiNH^+$
$C_2H_6N^+$	alanine	44.05	44.05	44	$SiNH_2^+$
CHS^+	cysteine	44.98	44.98	45	$SiNH_3^+$
$C_3H_6N^+$	lysine	56.05	56.05	56	Si_2^+
$CH_5N_3^+$	arginine	59.05	59.05	59	
$C_2H_6NO^+$	serine	60.04	60.06	60	
$C_2H_5S^+$	methionine	61.01	61.01	61	
$C_4H_6N^+$	proline	68.05	68.05	68	
$C_4H_5O^+$	threonine	69.03	69.04	69	
$C_4H_8N^+$	proline	70.07	70.07	70	
$C_3H_3O_2^+$	serine	71.01	71.02	71	
$C_4H_{10}N^+$	valine	72.08	72.09	72	
$C_2H_7N_3^+$	arginine	73.06	73.07	73	$Si(CH_3)_3^+$
$C_3H_8NO^+$	threonine	74.06	74.07	74	
$C_2H_6NS^+$	cysteine	76.02	76.03	76	
$C_5H_6N^+$	proline	80.05	80.05	80	
$C_4H_5N_2^+$	histidine	81.05	81.05	81	
$C_4H_6N_2^+$	histidine	82.05	82.07	82	
$C_5H_7O^+$	valine	83.05	83.06	83	
$C_5H_{10}N^+$	lysine	84.08	84.09	84	
$C_5H_{12}N^+$	leucine	86.10	86.10	86	
$C_3H_7N_2O^+$	asparagine	87.06	87.09	87	
$C_3H_6NO_2^+$	aspartic acid	88.04	88.04	88	
$C_4H_4NO_2^+$	asparagine	98.02	98.02	98	
$C_4H_{10}N_3^+$	arginine	100.09	100.09	100	
$C_4H_{11}N_3^+$	arginine	101.10	101.08	101	
$C_4H_8NO_2^+$	glutamic acid	102.06	102.06	102	
$C_4H_{10}NS^+$	methionine	104.05	104.06	104	
$C_7H_7O^+$	tyrosine	107.05	107.05	107	
$C_5H_8N_3^+$	histidine	110.07	110.08	110	
$C_5H_{10}N_3^+$	arginine	112.09	112.09	112	
$C_5H_9SO^+$	methionine	117.04	117.06	117	
$C_8H_{10}N^+$	phenylalanine	120.08	120.09	120	
$C_9H_8N^+$	tryptophan	130.07	130.07	130	
$C_9H_7O^+$	phenylalanine	131.05	131.05	131	
$C_9H_8O^+$	phenylalanine	132.06	132.06	132	
$C_8H_{10}NO^+$	tyrosine	136.08	136.08	136	
$C_9H_7O_2^+$	tyrosine	147.04	147.05	147	$Si_2(CH_3)_5O^+$
$C_{10}H_{11}N_2^+$	tryptophan	159.09	159.09	159	
$C_{11}H_8NO^+$	tryptophan	170.06	170.06	170	

Table S3 95% confidence limits for all the PCA plots

confidence limits	**Fig. 3b**	**Fig. 3d**	**Fig. S6b**	**Fig. S6d**	**Fig. S16b**
**BSA**	[0.022, 0.104]	[0.019, 0.091]	[0.701, 3.345]	[0.112, 0.533]	[0.041, 0.193]
**collagen**	[-0.265, -0.055]	[-0.322, -0.068]	[-1.608, -0.337]	[-0.320, -0.067]	[0.002, 0.007]
**fibronectin**	[-0.430, -0.090]	[-0.380, -0.080]	[-0.147, -0.031]	[-0.303, -0.064]	[-0.356, -0.075]
**laminin**	[0.040, 0.192]	[0.040, 0.192]	[-0.787, -0.165]	[0.006, 0.026]	[-0.138, -0.029]
**vitronectin**	[0.031, 0.147]	[0.033, 0.160]	[0.239, 1.138]	[0.060, 0.286]	[0.062, 0.294]
**water**	[0.053, 0.250]	[0.054, 0.259]	[-1.941, -0.407]	[-0.222, -0.046]	

confidence limits	**Fig. S12b**
**dried laminin**	[-0.468, -0.098]
**protected laminin**	[-0.235, -0.049]
**hydrated laminin**	[0.125, 0.596]
**dried vitronectin**	[-0.395, -0.083]
**protected vitronectin**	[-0.191, -0.040]
**hydrated vitronectin**	[0.145, 0.693]

Table S4 The relative abundance of PDMS in the liquid SIMS protein spectrum

PDMS relative ion abundance	**1**	**2**	**3**
**BSA**	1.91%	1.83%	1.88%
**collagen**	2.31%	2.55%	2.37%
**fibronectin**	1.04%	1.28%	1.15%
**laminin**	1.98%	2.20%	2.18%
**vitronectin**	2.31%	2.19%	2.07%
**water**	1.51%	1.82%	1.55%

Table S5 The XPS determined composition of BSA, laminin, and vitronectin films absorbed onto clean Si substrates

Protein	Carbon (at%)	Nitrogen (at%)	Oxygen (at%)	Si (at%)
BSA	36.0±2.2	1.5±0.3	32.0±0.7	30.6±1.3
Laminin	33.2±1.4	5.6±0.3	31.3±0.6	29.8±1.0
Vitronectin	36.3±4.2	3.4±0.2	31.1±0.9	29.2±3.1
Si control	17.1±1.8	0.0±0.0	38.4±0.6	44.4±1.2

MOVIE CAPTION

Movie S1 Merged 3D illustrations of selected positive amino acid fragments, positive water clusters, and negative water clusters from hydrated fibronectin film, hydrated laminin film, and pure water. Data were reconstructed from Region II in Fig. 1c.

REFERENCES

1. L. Yang, X.-Y. Yu, Z. Zhu, T. Thevuthasan and J. P. Cowin, *J. Vac. Sci. Technol., A*, 2011, **29**, 061101.
2. L. Yang, Z. Zhu, X.-Y. Yu, S. Thevuthasan and J. P. Cowin, *Anal. Methods*, 2013, **5**, 2515-2522.
3. X. Hua, M. J. Marshall, Y. Xiong, X. Ma, Y. Zhou, A. E. Tucker, Z. Zhu, S. Liu and X.-Y. Yu, *Biomicrofluidics*, 2015, **9**, 031101.
4. X. Hua, X.-Y. Yu, Z. Wang, L. Yang, B. Liu, Z. Zhu, A. E. Tucker, W. B. Chrisler, E. A. Hill, T. Thevuthasan, Y. Lin, S. Liu and M. J. Marshall, *Analyst*, 2014, **139**, 1609-1613.
5. J. Yu, Y. Zhou, X. Hua, Z. Zhu and X.-Y. Yu, *J. Visualized Exp.*, 2016, e53708.
6. J. J. Gray, *Curr. Opin. Struct. Biol.*, 2004, **14**, 110-115.
7. N. Xia, C. J. May, S. L. McArthur and D. G. Castner, *Langmuir*, 2002, **18**, 4090-4097.
8. M. S. Wagner, T. A. Horbett and D. G. Castner, *Langmuir*, 2003, **19**, 1708-1715.
9. J. Wald, C. Muller, M. Wahl, W. Hoth-Hannig, M. Hannig, M. Kopnarski and C. Ziegler, *Phys. Status Solidi A*, 2010, **207**, 831-836.
10. C. Bruning, S. Hellweg, S. Dambach, D. Lipinsky and H. F. Arlinghaus, *Surf. Interface Anal.*, 2006, **38**, 191-193.
11. O. D. Sanni, M. S. Wagner, D. Briggs, D. G. Castner and J. C. Vickerman, *Surf. Interface Anal.*, 2002, **33**, 715-728.
12. M. S. Wagner and D. G. Castner, *Langmuir*, 2001, **17**, 4649-4660.
13. S. Muramoto, D. J. Graham, M. S. Wagner, T. G. Lee, D. W. Moon and D. G. Castner, *J. Phys. Chem. C*, 2011, **115**, 24247-24255.
14. L. Yang, X.-Y. Yu, Z. Zhu, M. J. Iedema and J. P. Cowin, *Lab Chip*, 2011, **11**, 2481-2484.
15. L. Yang, Z. Zhu, X.-Y. Yu, E. Rodek, L. Saraf, T. Thevuthasan and J. P. Cowin, *Surf. Interface Anal.*, 2014, **46**, 224-228.
16. L. Baugh, T. Weidner, J. E. Baio, P. C. T. Nguyen, L. J. Gamble, P. S. Slayton and D. G. Castner, *Langmuir*, 2010, **26**, 16434-16441.
17. H. Wang, D. G. Castner, B. D. Ratner and S. Y. Jiang, *Langmuir*, 2004, **20**, 1877-1887.
18. J. E. Baio, T. Weidner, G. Interlandi, C. Mendoza-Barrera, H. E. Canavan, R. Michel and D. G. Castner, *Journal of vacuum science & technology. B, Microelectronics and nanometer structures : processing, measurement, and phenomena : an official journal of the American Vacuum Society*, 2011, **29**, 4D113-114D113.
